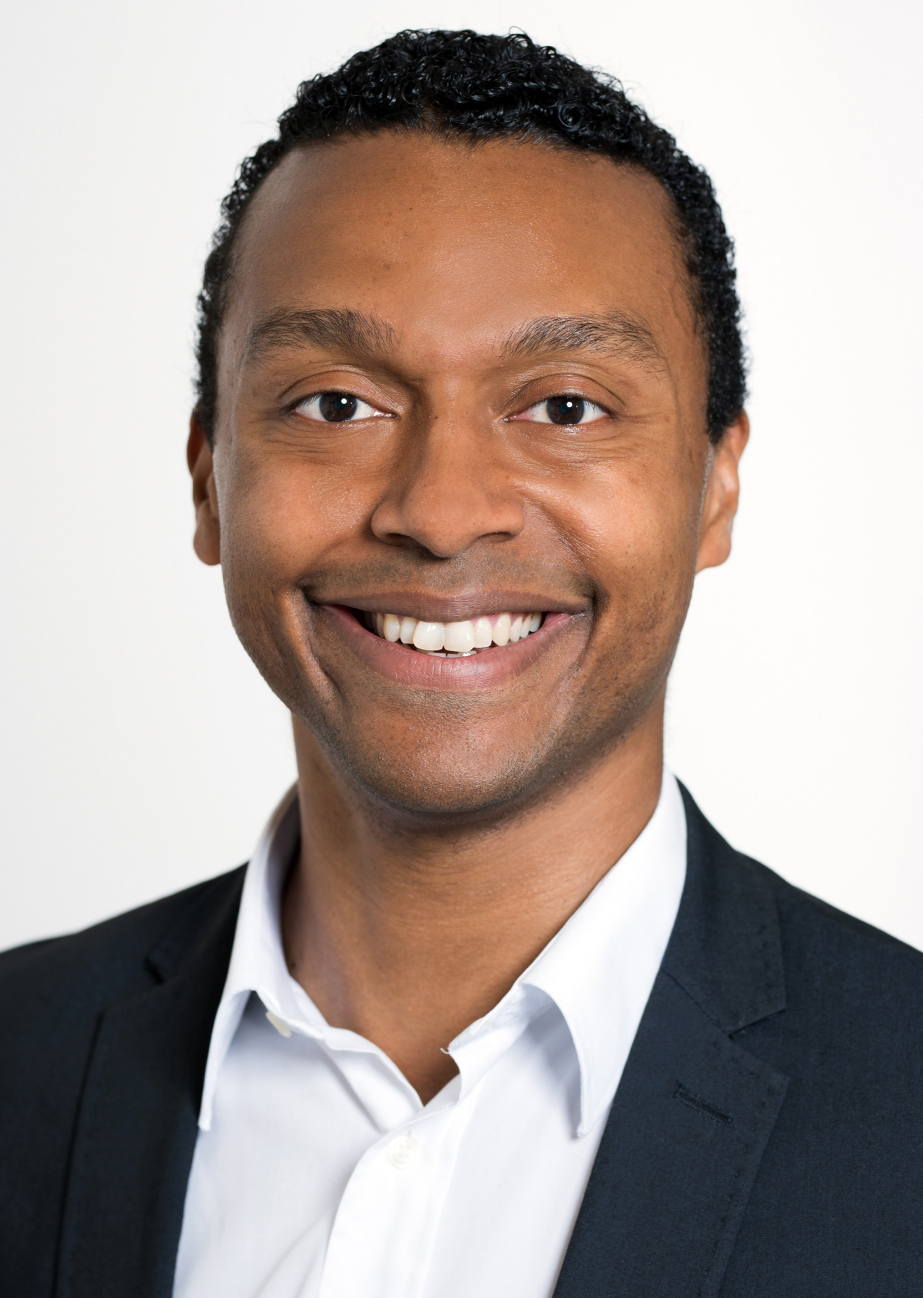# Challenges and Breakthroughs in Selective Amide Activation

**DOI:** 10.1002/ange.202212213

**Published:** 2022-11-02

**Authors:** Minghao Feng, Haoqi Zhang, Nuno Maulide

**Affiliations:** ^1^ Faculty of Chemistry Institute of Organic Chemistry University of Vienna Währinger Straße 38 1090 Vienna Austria; ^2^ Christian-Doppler Laboratory for Entropy-Oriented Drug Design Josef-Holaubek-Platz 2 1090 Vienna Austria

**Keywords:** Amide Activation, Amide Functionalisation, Electrophilic Activation, Synthetic Methods, Transition-Metal Catalysis

## Abstract

In contrast to ketones and carboxylic esters, amides are classically seen as comparatively unreactive members of the carbonyl family, owing to their unique structural and electronic features. However, recent decades have seen the emergence of research programmes focused on the selective activation of amides under mild conditions. In the past four years, this area has continued to rapidly develop, with new advances coming in at a fast pace. Several novel activation strategies have been demonstrated as effective tools for selective amide activation, enabling transformations that are at once synthetically useful and mechanistically intriguing. This Minireview comprises recent advances in the field, highlighting new trends and breakthroughs in what could be called a new age of amide activation.

## Graphical abstract

Research on the selective activation of amides has flourished over the last decades. In the past four years, this area has become a rapidly developing domain. This Minireview aims to highlight the breakthroughs in amide activation achieved since 2018, with a focus on significant advances in electrophilic and transition‐metal‐catalysed amide activation, as well as other strategies on amide activation and functionalisation.

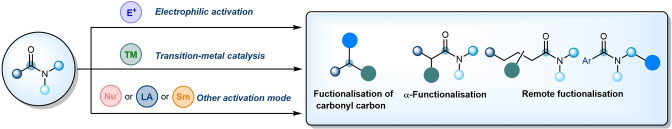

## 1 Introduction

**Scheme 1:**
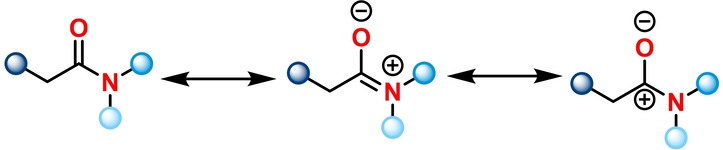
Illustration of amidic resonance structures.

Carbonyl groups are among the most common functionalities in organic molecules and methodologies; allowing their selective functionalisation while concomitantly enabling an increase in molecular complexity are in consistently high demand. In contrast to acyl halides, anhydrides, ketones and esters, amides have historically been considered to be comparatively unreactive. The unique delocalisation of the lone pair of electrons at nitrogen on the π* system,[^1^, ^2^, ^3^] also known as amidic resonance, is responsible for an increased stabilisation of the electrophilic carbonyl carbon (Scheme 1).^[4]^ This difference in reactivity has led to the perception that amides are significantly less useful functional handles than their ester and acyl halide counterparts. Nonetheless, amides are among the most frequently encountered moieties in functional molecules such as pharmaceutical compounds, bioactive natural products and polymeric materials. Methodologies towards amide activation have proven to facilitate the synthesis of corresponding amide‐containing functional molecules and their analogues. Moreover, highly selective amide activation can also be a valuable tool for late‐stage drug modification, thereby benefiting drug discovery and other fields of medicinal chemistry.

Accordingly, synthetic chemists have devoted tremendous efforts to the selective activation of amides since the 19^th^ century. A comprehensive review on amide activation prior to 2018 was presented by the Maulide group.^[5]^ Work in this fast‐developing research field aims to deploy the carboxamide moiety in reactions of diverse nature, where functionalisation occurs either at the carbonyl carbon or at neighbouring positions.^[6]^ Particularly worthy of mention is the trend for such processes to be chemoselective for the carboxamide functionality, even when other, traditionally more reactive carbonyl moieties (such as esters or ketones) are present.

In very recent years, research in this field has stepped into a new age with ever more varied activation strategies and reaction partners. This Minireview highlights breakthroughs in amide activation since 2018, with a focus on significant advances in electrophilic^[5]^ and transition‐metal‐catalysed amide activation.[^7^, ^8^] Other strategies initiated by nucleophilic addition^[9]^ or SmI_2_/Sm‐redox processes shall be discussed as well.

## 2 Electrophilic Amide Activation

**Scheme 2:**
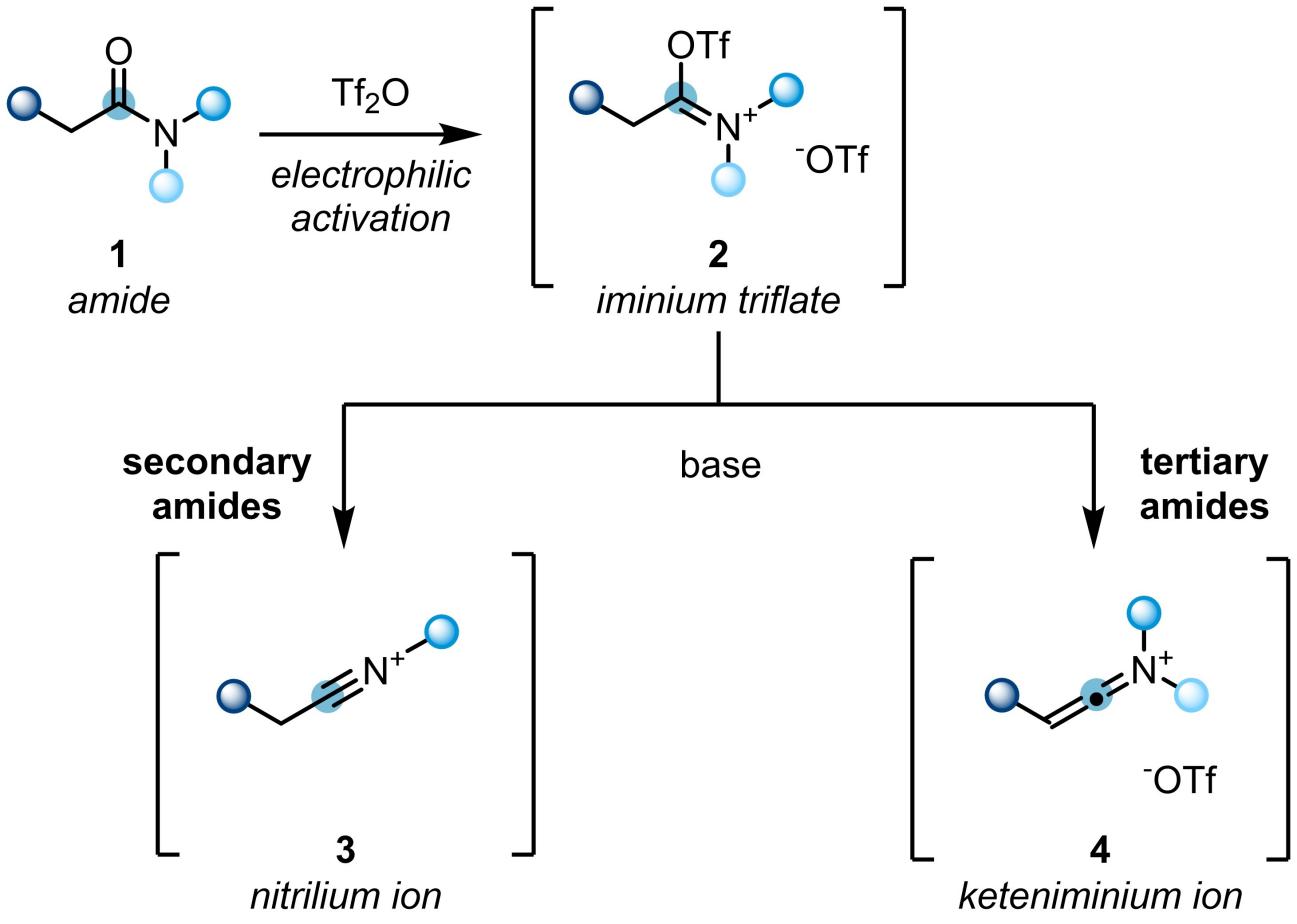
Electrophilic activation of tertiary and secondary amides with Tf_2_O.

The functionalisation of carbonyl derivatives by nucleophilic addition is considered as textbook toolbox chemistry. However, the use of unmodified amides in the same transformations is often challenging, owing to their intrinsic low electrophilicity. Hence, a pre‐activation is usually required. Electrophilic amide activation can be achieved with oxophilic reagents, such as phosgene, PCl_5_, oxalyl chloride and triflic anhydride (Tf_2_O). The deployment of Tf_2_O has been established as a powerful strategy,^[5]^ due to its comparably low toxicity, mild reaction protocol and high chemoselectivity. Notably, the mode of reactivity is highly dependent on the nature of the amide, with secondary and tertiary amides showing differences (Scheme 2).[^10^, ^11^] Initially, the treatment of amides **1** with Tf_2_O leads to iminium triflates **2**. Upon further deprotonation, usually mediated by a pyridine base, these are converted to highly electrophilic nitrilium ions **3** for secondary amides, or reactive keteniminium ions **4** in case of their tertiary counterparts. These in situ generated reactive species then set the stage for further transformations.

### 2.1 Ipso‐Activation of Amides

**Scheme 3:**
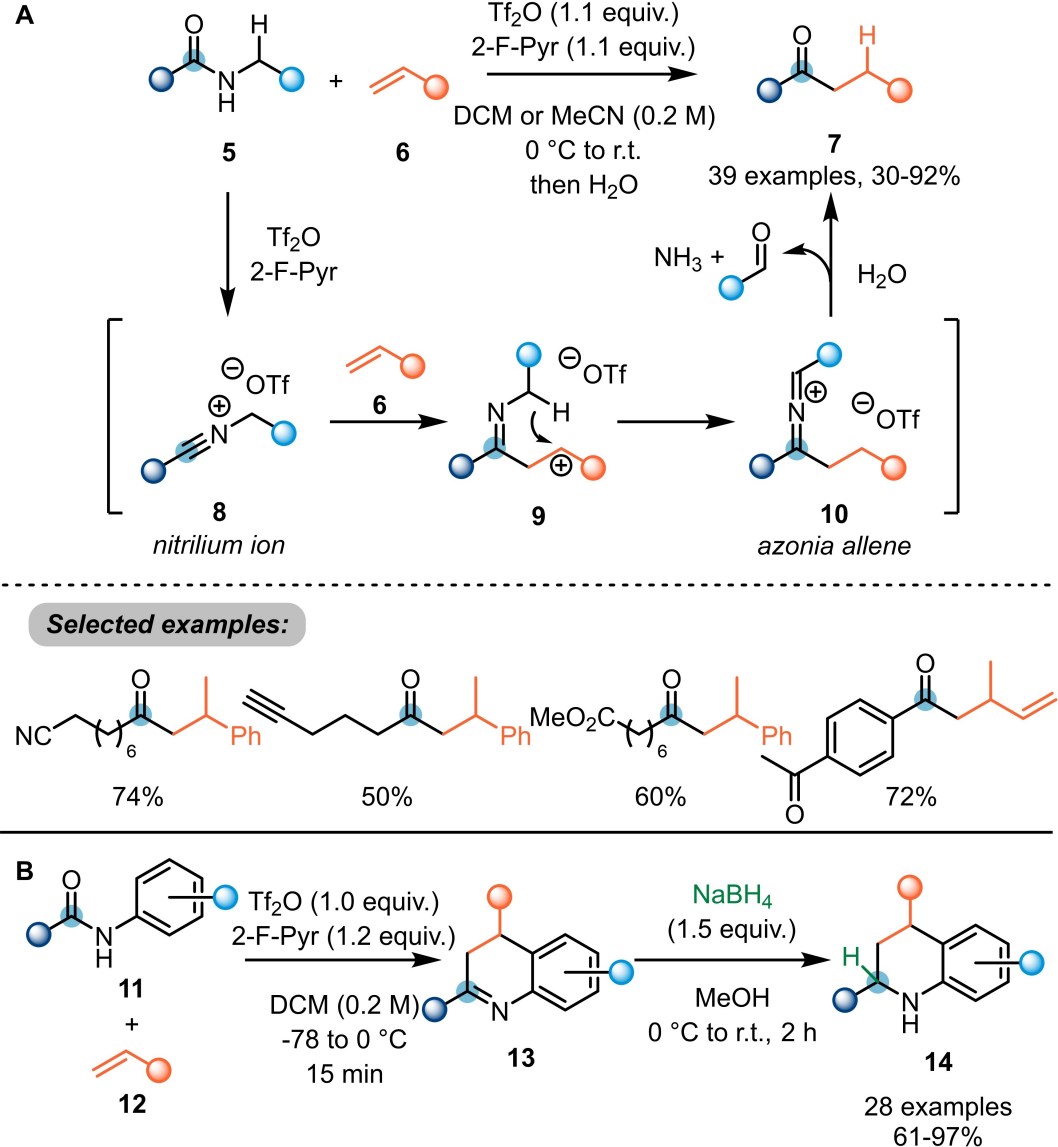
A) Hydroacylation of alkenes using activated secondary amides. B) [4+2]‐Cycloaddition forming annulated nitrogen heterocycles by reaction of *N*‐aryl amides with alkenes to access 3,4‐dihydroquinolines **14**.

As can be deduced from the above section, electrophilic amide activation allows considerable increase in reactivity at the *ipso* position towards nucleophiles. For instance, combining the increased electrophilicity of activated secondary amides (**5**→**8**) with the inherent nucleophilicity of terminal alkenes **6**, Maulide et al. were able to develop a highly chemoselective alkene hydroacylation reaction to access ketones **7** under transition‐metal‐free conditions (Scheme 3A).^[12]^ Herein, initial electrophilic activation of the amide with triflic anhydride leads to the formation of nitrilium ion **8**. This species is prone to attack by an alkene (**6**) to furnish intermediate **9**, ideally suited for intramolecular 1,5‐hydride shift. The resulting azonia allene **10** is cleaved by aqueous work up to yield the desired ketone **7**. Notably, the reaction tolerated a wide range of functional groups, including several carbonyl derivatives classically considered to be more reactive (cf. “Selected examples” in Scheme 3A).

In a related approach, Huang et al. reported a [4+2]‐annulation with *N*‐aryl amides **11** and alkenes **12** to afford 3,4‐dihydroquinolines **13** (Scheme 3B).^[13]^ The authors also reported that a reductive work up is able to directly yield valuable tetrahydroquinolines **14** in high yields.

**Scheme 4:**
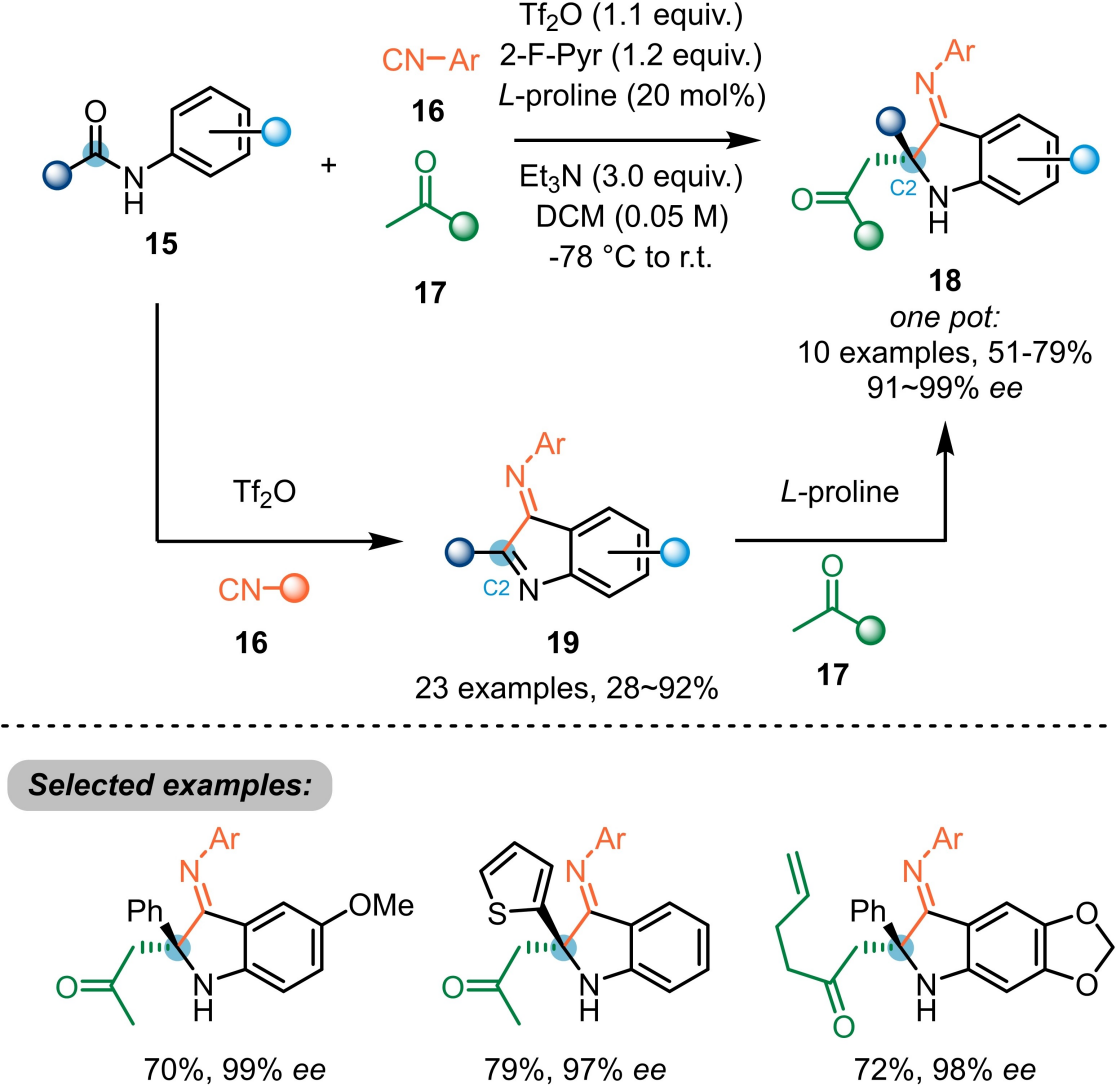
Enantioselective dual‐functionalisation of *N*‐aryl secondary amides **15** with triflic anhydride and a chiral organocatalyst.

Huang et al. further investigated the activation of secondary *N*‐aryl amides **15** by nucleophilic attack of isocyanide **16** (Scheme 4),^[14]^ ultimately leading to 2‐substituted 3‐iminoindoles **19**. Additionally, the authors exploited the electrophilic nature of the newly formed heterocycle for an organocatalysed, asymmetric Mannich‐type addition of ketones **17**. Despite the presence of two imine groups, a highly enantioselective functionalisation at the C2 position yielding 3‐iminoindolines **18** could be achieved in a one‐pot fashion.

**Scheme 5:**
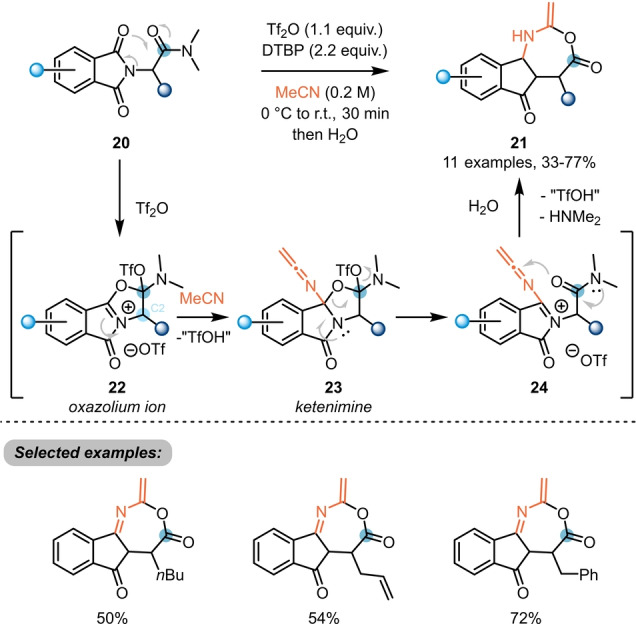
Synthesis of unusual 7‐membered fused heterocycle **21** from α‐phthalimido‐amides **20**. DTBP: 2,6‐di‐*tert*‐butylpyridine.

Electrophilic activation at the *ipso*‐position of amides was further explored for the synthesis of novel classes of heterocycles, once more showcasing the chemical versatility of the evoked intermediates. In 2020, Maulide et al. reported the use of triflic anhydride for the synthesis of unusual 7‐membered fused heterocycles **21** from α‐phthalimido‐amides **20** (Scheme 5).^[15]^ From a mechanistic point of view, electrophilic activation takes place at the amide, which is more nucleophilic than the tethered imide. This activation is thought to trigger intramolecular attack by the imide, resulting in formation of an oxazolinium ion **22**. Notably, experiments including an enantioenriched C2 position indicated that this process does not involve a keteniminium intermediate. Subsequent attack by acetonitrile, used as the solvent of this reaction, was then proposed to yield ketenimine **23**, before a two‐step ring expansion closed the 7‐membered ring, yielding **21** after isomerisation and hydrolysis.

**Scheme 6:**
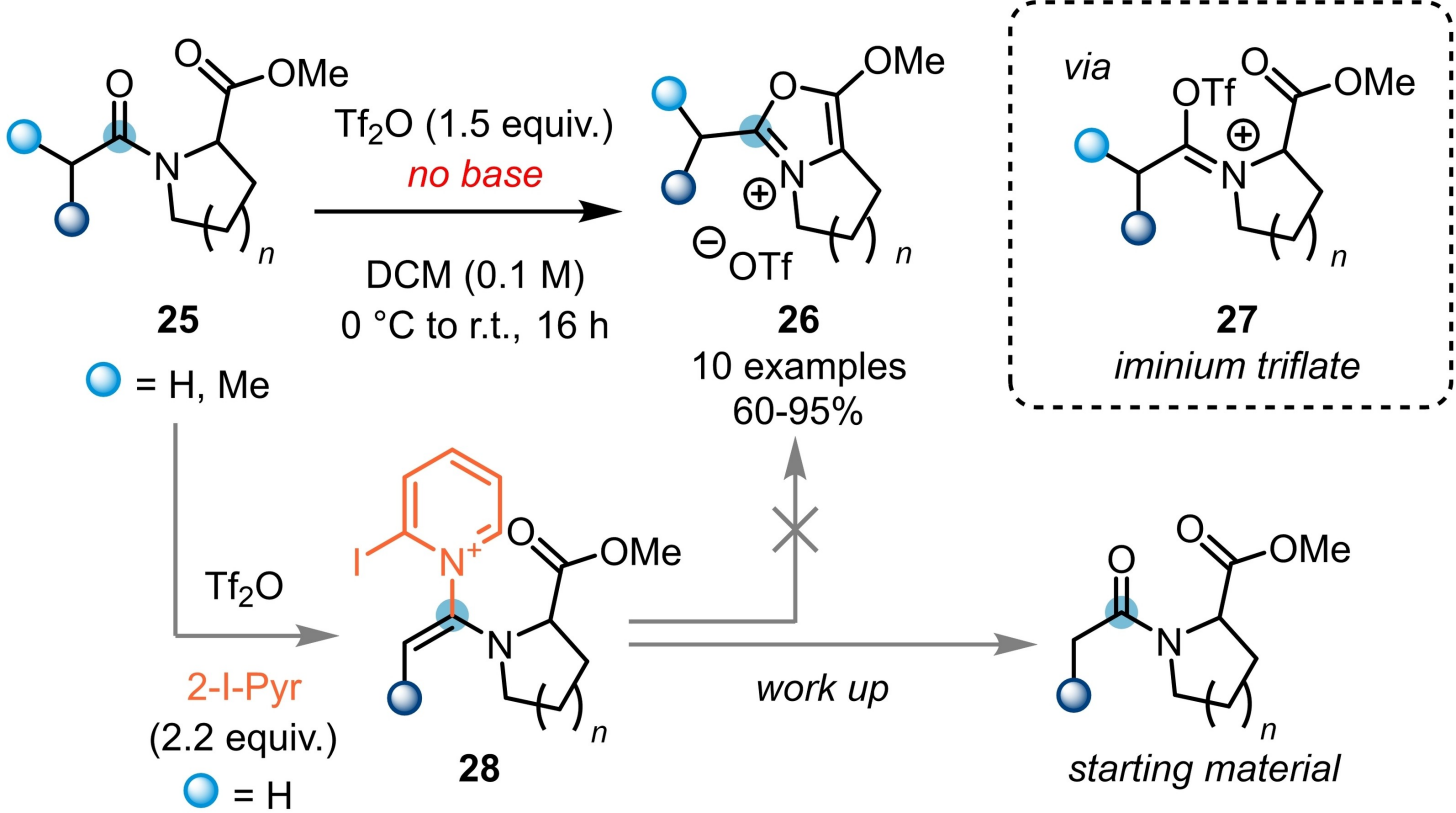
Synthesis of bicyclic alkoxyoxazolium salts **26** from amides **25** in the absence of base.

The effect of the substitution pattern of the pyridine bases used in classical triflic‐anhydride‐mediated amide activation is often not fully understood.^[16]^ For this reason, a range of substituted pyridines is commonly surveyed when optimising reactions involving electrophilic amide activation.[^12^, ^15^] However, in certain circumstances, the presence of base can even prohibit the desired reactivity. This was observed by Maulide et al. during their endeavour to synthesise alkoxyoxazolium salts **26** (Scheme 6), resulting from intramolecular capture of the electrophilic intermediate by a proximal ester moiety.^[17]^ In this work, control experiments showed that amides **25** with easily accessible α‐protons are prone to be deprotonated and attacked by the pyridine base. The formed intermediate **28** inhibits the attack of the ester, leading to starting material recovery after aqueous work up. In contrast, under exclusion of the base, iminium triflate **27** is the predominant active species, and facilitates the desired cyclisation event.

**Scheme 7:**
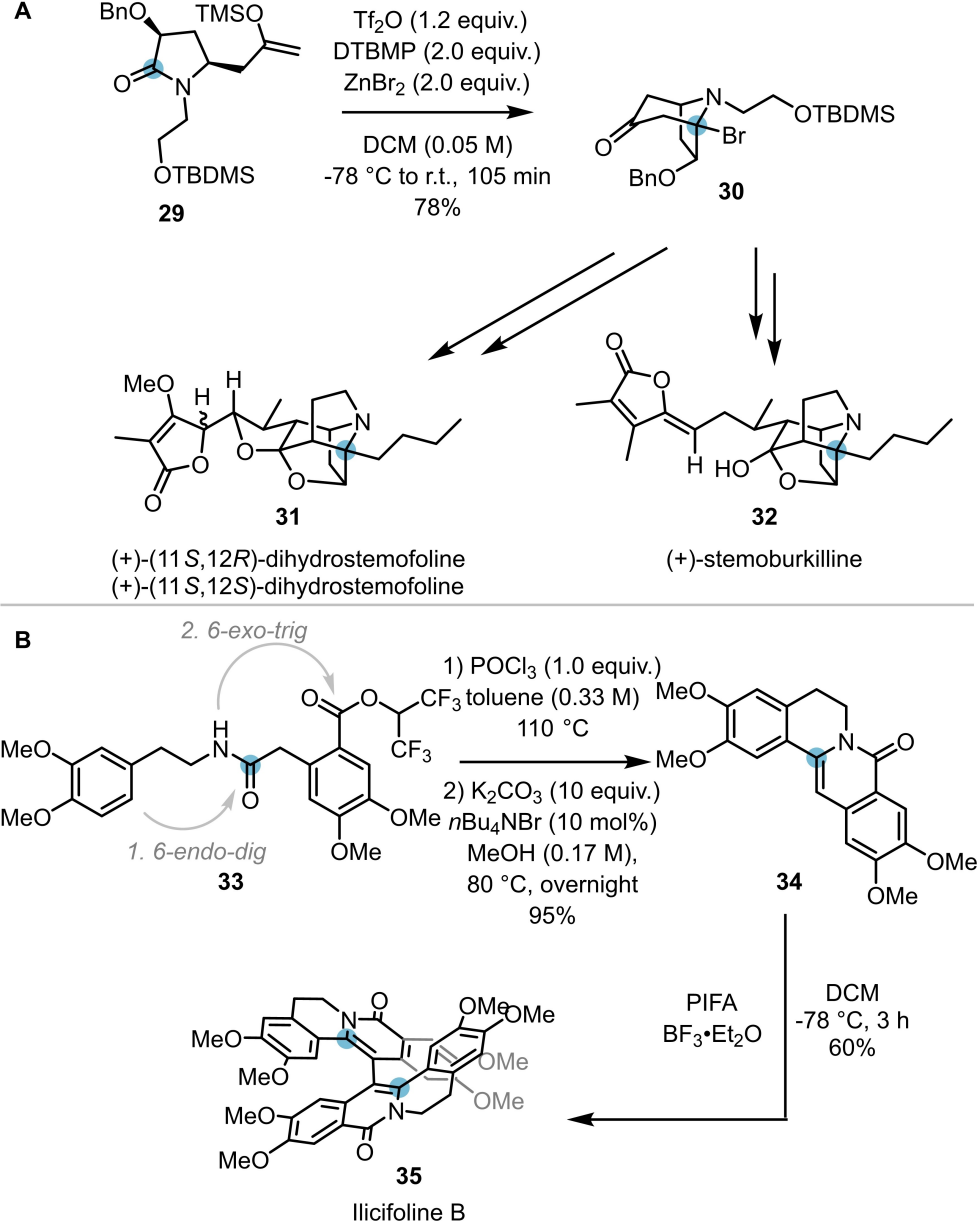
Electrophilic amide activation in total synthesis. A) Total synthesis of (+)‐stemofoline (**31**) via a two‐step keto–lactam cyclisation–bromination cascade. B) Total synthesis of ilicifoline B (**35**) via POCl_3_‐mediated amide activation.

Due to its high selectivity and chemical versatility, the intramolecular capture of intermediates resulting from electrophilic amide activation has also featured in the total syntheses of several natural products. In 2020, Huang et al. exploited the intramolecular attack of a silyl enol ether (**29**) on an activated amide for the synthesis of the core structure (**30**) of stemofoline (**31**) and stemoburkiline (**32**) (Scheme 7A).^[18]^

A further example was reported by Christmann and co‐workers, who successfully applied the amide activation approach to their concise total synthesis of ilicifoline B **35** (Scheme 7B).^[19]^ Herein, upon activation of amide **33** with POCl_3_ and subsequent addition of potassium carbonate and a phase transfer catalyst, tetracyclic scaffold **34** was obtained in excellent yield (affording the natural product after final oxidative dimerisation). Notably, this intriguing reaction proceeds via amide activation and an *endo‐dig* cyclisation, followed by an ester activation mediated *exo‐trig* cyclisation step.

### 2.2 α‐Functionalisation of Amides

The α‐position of carbonyls possesses intrinsic nucleophilic character that can be exploited for functionalisation through deprotonation by a strong base (e.g. ketone p*K*_a_≈24). Amides are no exception, but exhibit low α‐acidity (p*K*_a_≈35) due to the comparatively weak electron‐withdrawing effect of the amide carbonyl. In contrast to the well‐established methods for polarity reversal of other carbonyl derivatives, the concept of amide umpolung has only recently been extensively explored.^[20]^ Following a previously reported method for amide umpolung using pyridine *N*‐oxide derivatives, developed by Maulide et al.,[^21^, ^22^] further investigation of the potential of this reaction has been undertaken.

**Scheme 8:**
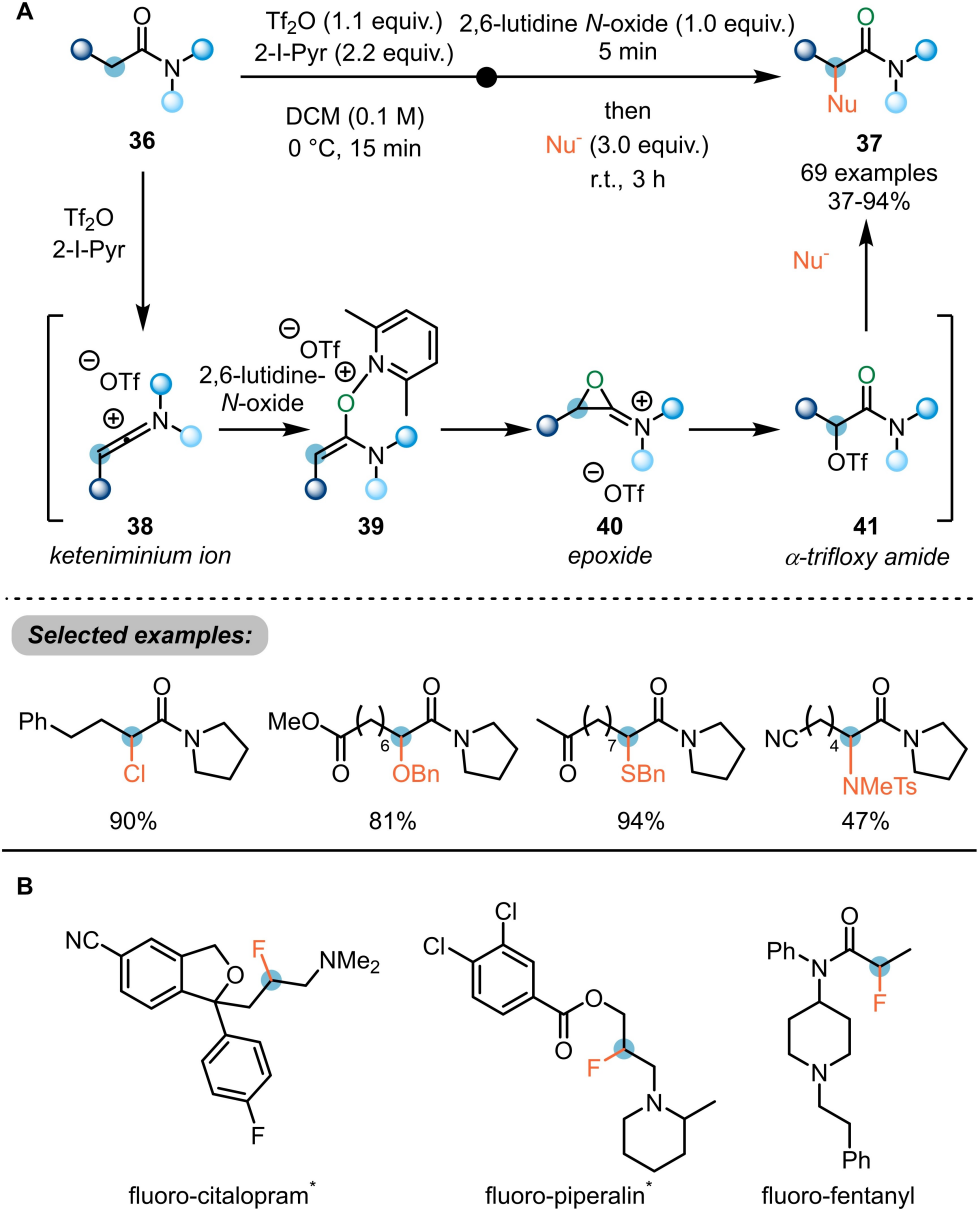
α‐Umpolung of amides with pyridine *N*‐oxides. A) α‐Functionalisation of amides with different nucleophiles and the proposed mechanism. B) Selected examples of fluorinated drug derivatives synthesised using the α‐fluorination of amides developed by Maulide et al. *Compounds were synthesised by reduction of the corresponding amides.

In the event, a range of heteroatom‐based nucleophiles can be coupled to the α‐position of amides (**36**) (Scheme 8A).^[23]^ This work moreover presented new insights into the reaction mechanism: while initial evidence had suggested the direct nucleophilic attack on the enolonium species **39**, resulting from addition of a pyridine *N*‐oxide derivative to the in situ generated keteniminium ion **38**, in‐depth experimental and computational studies rather supported the generation of an epoxide intermediate **40** which undergoes interception by the triflate counterion to yield the α‐trifloxy amide **41**. Indeed, the latter was proven to be an intermediate of this process by independent synthesis and subjection to the reaction conditions. Suitable nucleophiles for displacement of the triflate include halides, alcohols, thiols and tosyl amides. As is common to electrophilic amide activation, functional group tolerance is high with esters, ketones, nitriles and alkenes all being allowed in substrate structure. Remarkably, this method was also successfully employed in the synthesis of α‐fluoro amides employing common fluoride salts as nucleophiles (Scheme 8B). Given the high basicity of fluoride, this result is noteworthy. It opened a door for drug optimisation in medicinal chemistry, as shown by the authors with the ready preparation of hitherto unknown fluoro‐citalopram and investigation of its biological properties.^[24]^

**Scheme 9:**
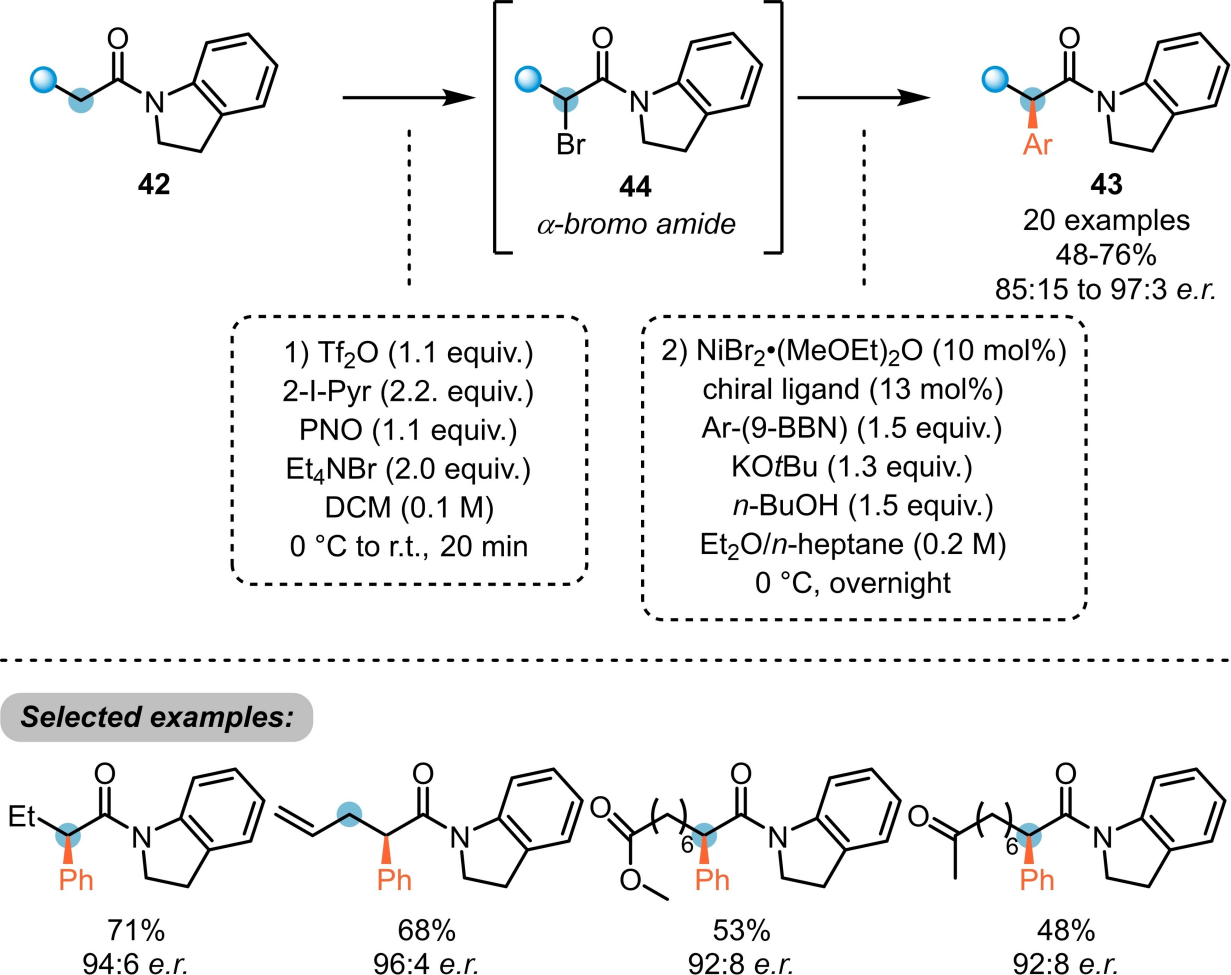
Amide umpolung enables Ni‐catalysed asymmetric α‐arylation of amides **42**.

The deeper understanding of the reaction mechanism and its intermediates enabled Maulide et al. to further develop an enantioselective C−C bond formation process by umpolung (Scheme 9).^[25]^ Herein, the direct formation of α‐bromo amides (**44**), via the corresponding α‐oxytriflated intermediates (not shown) enabled an asymmetric Ni‐catalysed Suzuki cross‐coupling with arylboronates, yielding chiral α‐arylated amides **43**.

**Scheme 10:**
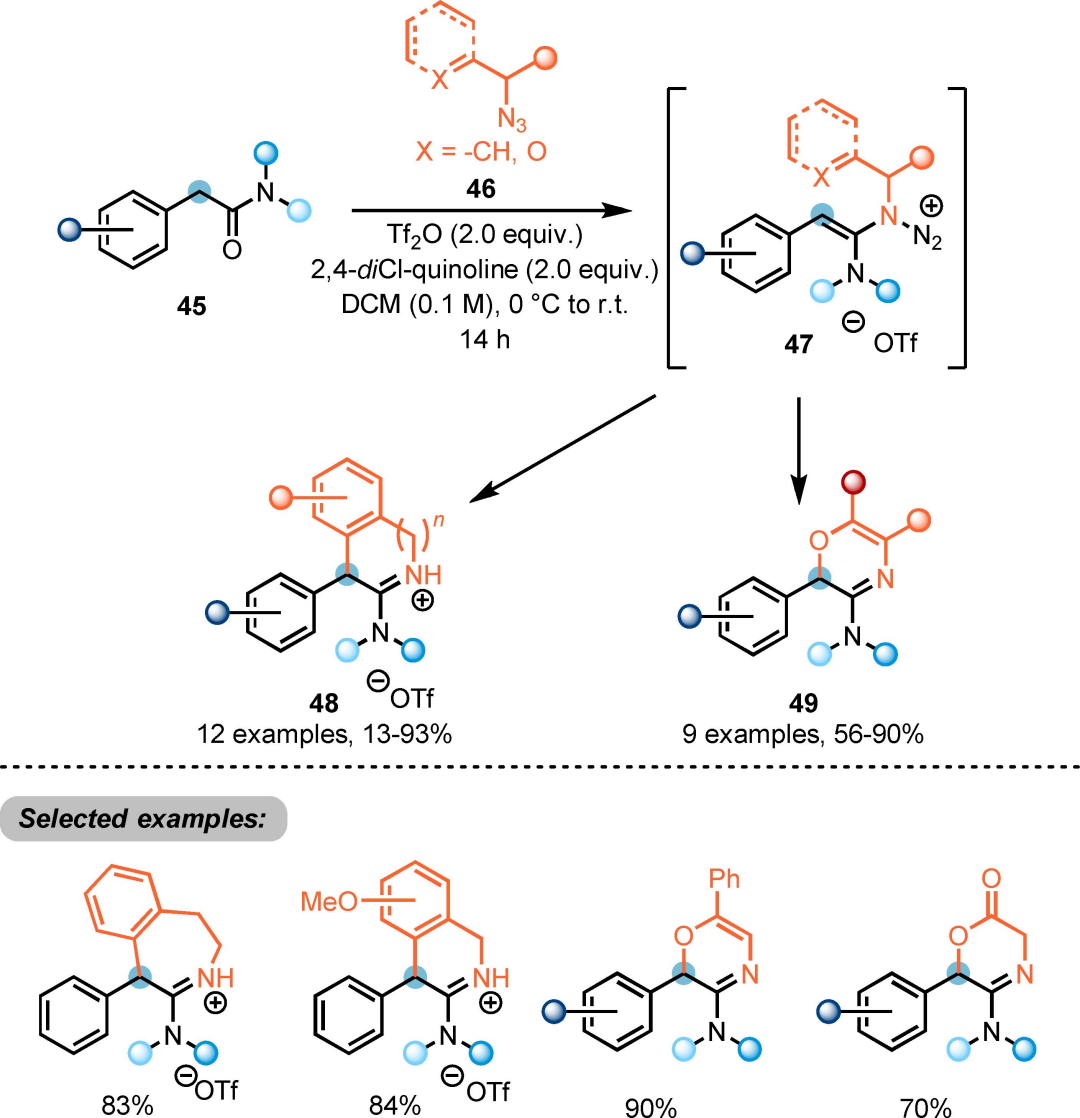
Synthesis of nitrogen‐containing heterocycles **48** and **49** via azide‐mediated umpolung of activated amides.

Maulide also reported the use of alkyl azides (**46**) in the synthesis of heterocycles (Scheme 10).^[26]^ Upon electrophilic activation of α‐arylated acetamides **45** with triflic anhydride, interception of the keteniminium ion by an azide **46** forms the intermediate **47**. Enabled by the extrusion of nitrogen gas, intramolecular attack at the electrophilic α‐carbon leads to formation of cyclised amidinium triflates **48** or oxazines **49**.

**Scheme 11:**
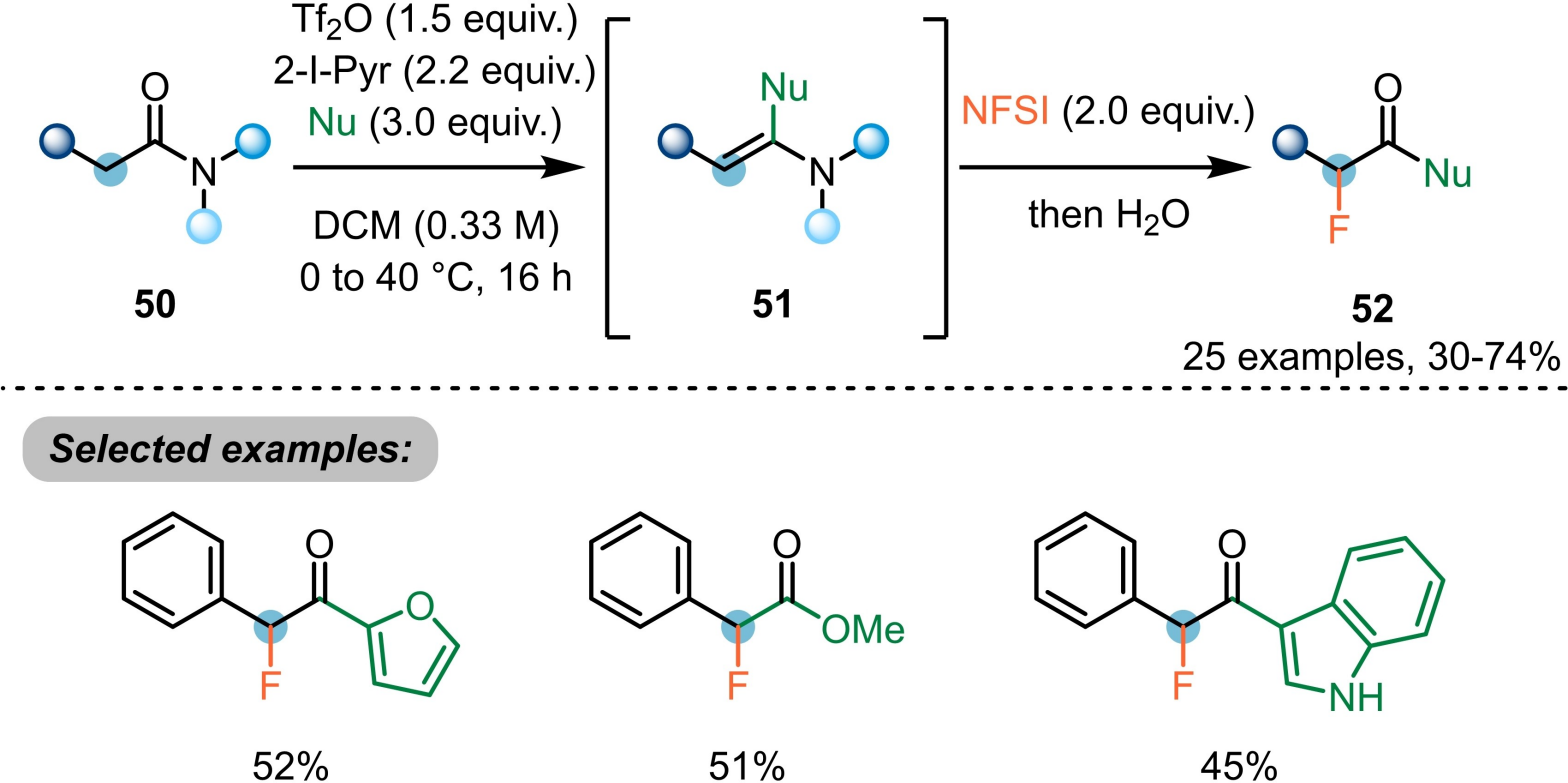
Synthesis of α‐fluorinated carbonyls **52** by double electrophilic amide activation.

Nucleophilic attack at the activated carbonyl position of amides can also facilitate α‐functionalisation without relying on umpolung. For example, Evano et al. have shown that the activated intermediates **51**, derived from **50**, can be brought to reaction with electrophilic fluorine sources to deliver α‐fluorocarbonyl derivatives **52** (Scheme 11).^[27]^ Heterocycles (furan and indoles) were well‐tolerated.

**Scheme 12:**
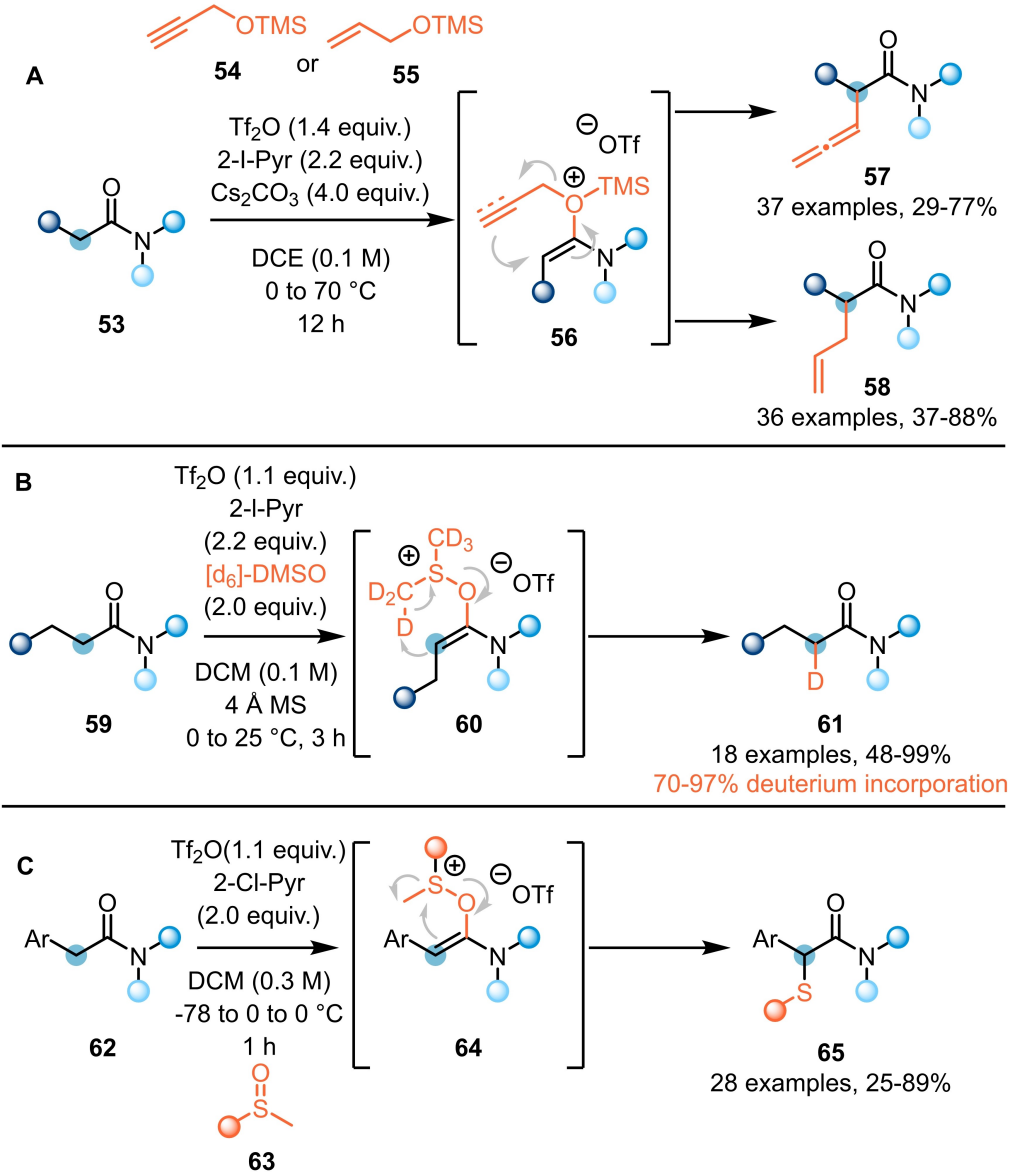
α‐Functionalisation of amides through rearrangement process. A) α‐Allenylation and α‐allylation via Claisen rearrangement. B) α‐Deuteration of α,β‐saturated amides with [d_6_]‐DMSO. C) α‐Sulfidation via sulfoxide rearrangement.

Sigmatropic rearrangements can also be employed to enable α‐functionalisation of amides (**53**). Recently, Liang et al. reported the synthesis of α‐allenyl (**57**) and α‐allyl amides (**58**) using silylated propargylic (**54**) and allylic alcohols (**55**), respectively (Scheme 12A).^[28]^ Therein, following formation of the reactive keteniminium intermediate, oxygen attack followed by desilylation forms a system **56** prone to [3,3]‐sigmatropic rearrangement (Claisen‐type). Treating activated amides with deuterated dimethyl sulfoxide ([d_6_]‐DMSO), Maulide et al. were able to selectively mono‐deuterate the α‐position (Scheme 12B).^[29]^ The process was proposed to proceed via a retro‐ene reaction of intermediate **60** and delivered the desired product **61** with high degrees of deuteration. Simultaneously, Movassaghi et al. reported the use of sulfoxides **63** for the synthesis of α‐sulfenylated amides **65** (Scheme 12C).^[30]^ While this process was initially found to only be applicable to α‐aryl acetamides, with aliphatic amides providing the retro‐ene products shown in Scheme 12B, alteration of the predominant mechanistic pathway by use of an additional electrophilic activator rendered the process general.

### 2.3 Remote Functionalisation of Amides

**Scheme 13:**
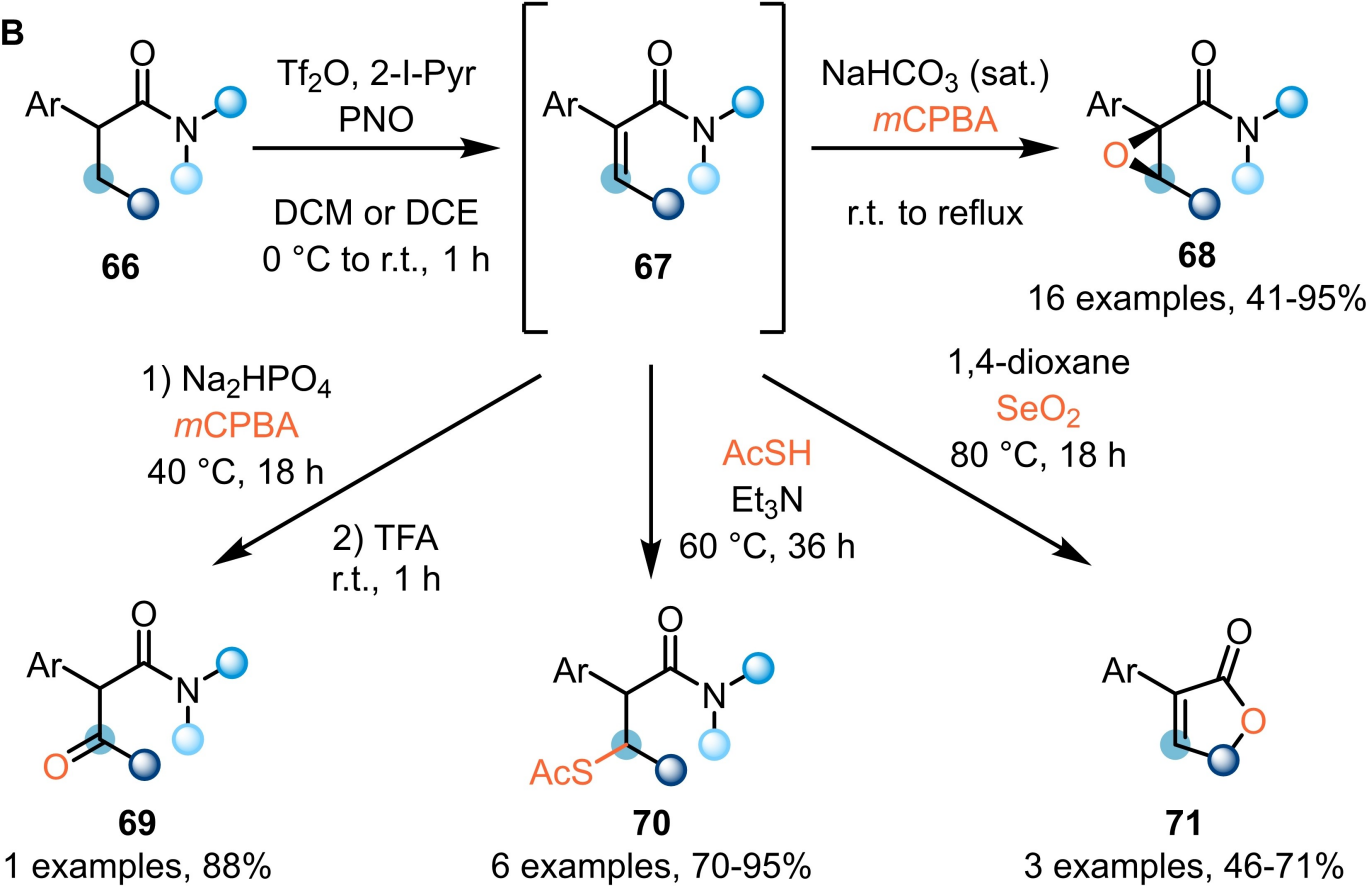
β‐Functionalisation of α‐branched amides enabled by transient dehydrogenation.

**Scheme 14:**
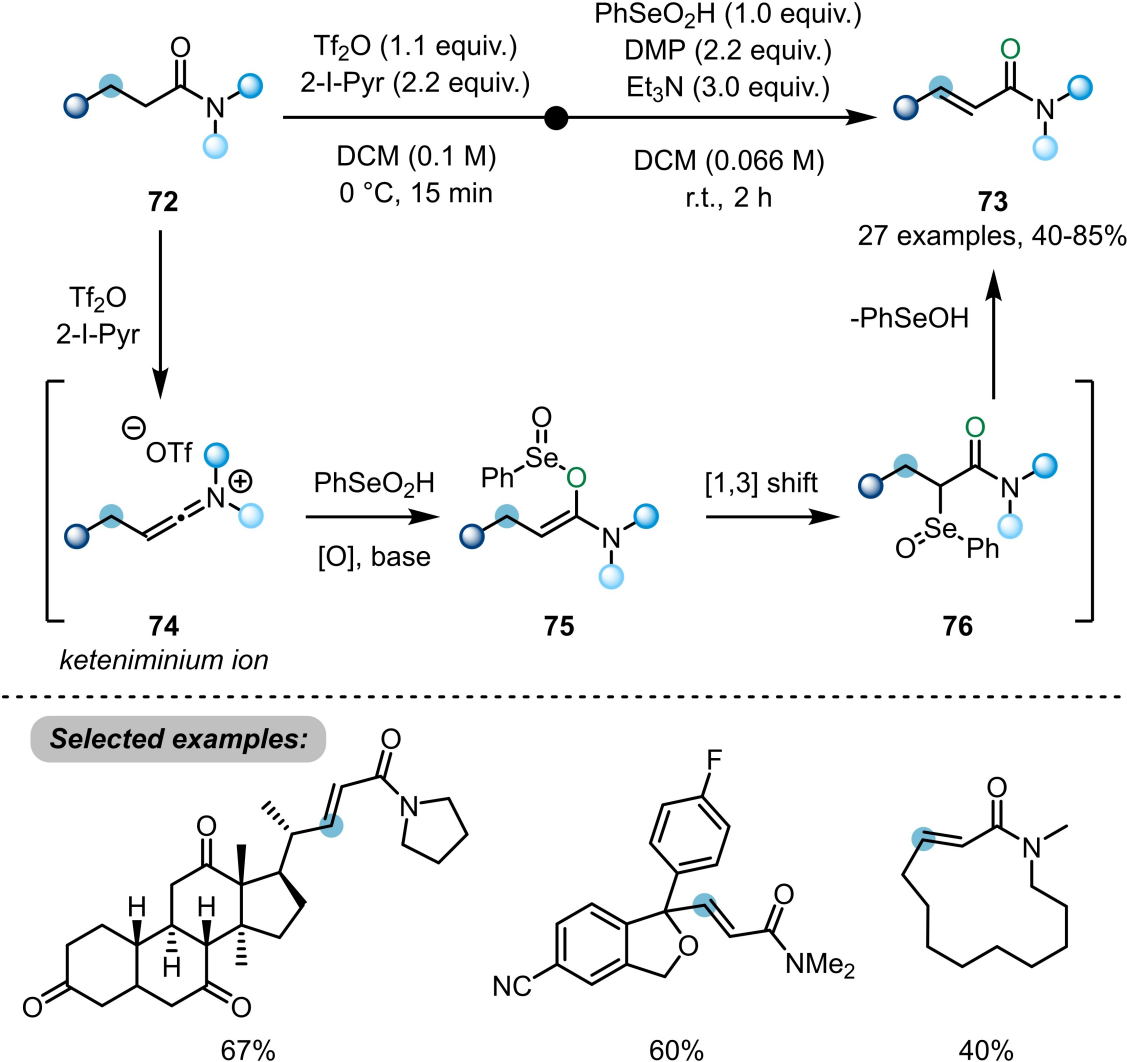
Selenium‐mediated α,β‐dehydrogenation of aliphatic amides.

While electrophilic amide activation was initially employed for functionalisation of the amide carbonyl, and later developments have largely targeted α‐functionalisation, most recently, the focus has shifted to the transformation of more remote C−H bonds. In this sense, Maulide et al. further demonstrated the versatility of electrophilic amide activation by developing methods for the α,β‐dehydrogenation of saturated amides **66** and **72** (Schemes 13 and 14).

Previous work by Ghosez had already hinted at the possibility of using *N*‐oxides for the α,β‐dehydrogenation of α‐branched amides.^[19]^ Based on this report, Maulide et al. were able to develop a general and direct oxidation protocol for the transformation of α‐branched‐α‐aryl amides **66** into the corresponding α,β‐unsaturated products **67** (Scheme 13).^[31]^ Remarkably, only the *Z*‐olefins were obtained from this transformation, and the synthetic utility of this class of compounds was demonstrated in a series of one‐pot derivatisations, including epoxidation (**68**), β‐oxidation (**69**), conjugate addition (**70**), and cyclisation to yield butenolides (**71**).

In order to find a reliable protocol for dehydrogenation of unbranched amides, Maulide turned to alternative reaction conditions and, notably, a different oxidant (Scheme 14).^[32]^ After formation of keteniminium ion **74**, nucleophilic addition of benzeneseleninic acid, in presence of an oxidant required to funnel a range of reaction pathways into the same product, leads to intermediate **75**. This species is capable of undergoing a [1,3]‐rearrangement to form α‐selenated amide **76**. Upon in situ elimination, it forms the α,β‐unsaturated amides **73**. Once more, this transformation can be achieved under mild conditions and with high functional group tolerance, preserving other carbonyl functionalities, such as esters and ketones, and was successfully applied to amide‐capped derivatives of drug candidates.

**Scheme 15:**
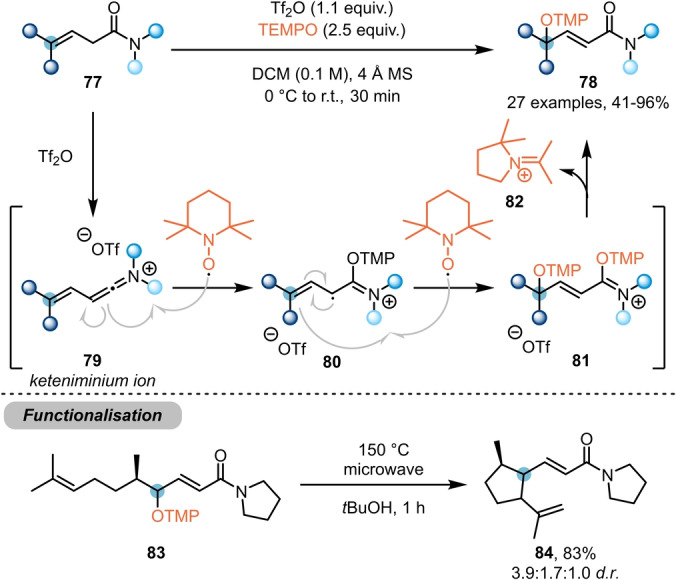
TEMPO‐mediated γ‐functionalisation of β,γ‐unsaturated amides **77**.“

Shifting focus even further from the amide carbonyl, the Maulide group developed a method for γ‐functionalisation of β,γ‐unsaturated amides **77** (Scheme 15).^[33]^ Herein, TEMPO ((2,2,6,6‐tetramethylpiperidin‐1‐yl)oxyl) was employed, instead of the previously used *N*‐oxides, thereby evoking a radical pathway. Mechanistically, the reaction is thought to proceed by TEMPO attack on the conjugated keteniminium ion **79**, generating radical intermediate **80**. Further interception of this species by a second equivalent of TEMPO leads to intermediate **81**, and subsequent fragmentation delivers the γ‐functionalised product **78** and the ring‐contracted iminium ion **82** (which was isolated after being computationally predicted in a previous study).^[34]^ The propensity of these systems towards forming allylic radicals was further demonstrated by subjecting **83** to elevated temperatures, forming **84** after radical 5‐*exo*‐*trig* cyclisation.

**Scheme 16:**
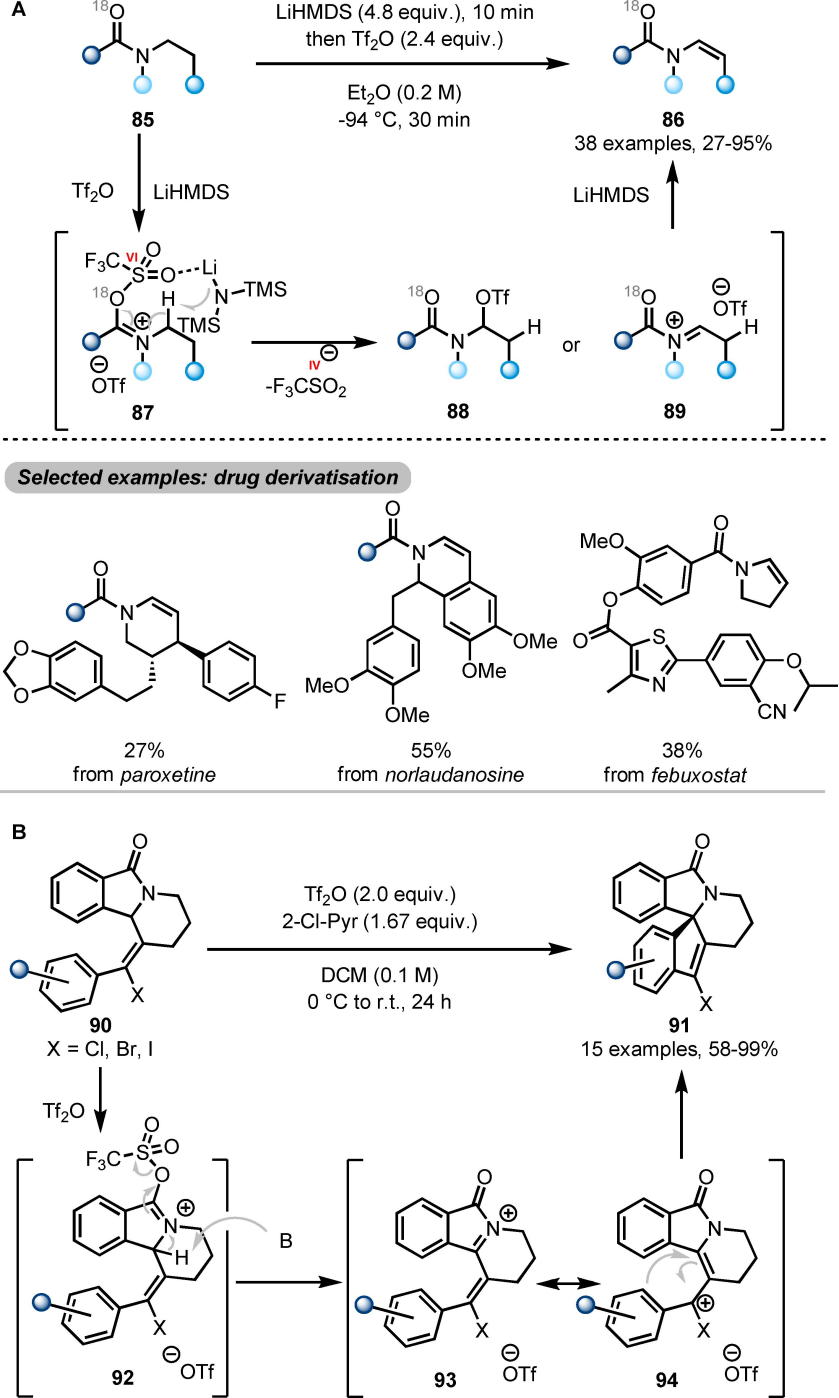
Remote functionalisation of activated *N*‐alkyl amides. A) Synthesis of enamides **86** by LiHMDS‐mediated dehydrogenation of amides. B) Synthesis of spirocyclic isoindolinones **91** via amide activation and halo‐Nazarov‐type cyclisation.

The work shown above reveals how electrophilic amide activation can lead to functionalisation of the “carbonyl side” (*ipso*‐, α‐, β‐, γ‐positions) of the amide precursors. In contrast, manipulations on the “nitrogen side” are rarely explored. An exception was recently published by Maulide et al. employing the unusual combination of Tf_2_O with a strong base (LiHMDS, lithium bis(trimethylsilyl)amide) to form enamides **86** (Scheme 16A) by a direct N‐dehydrogenation.^[35]^ From a sequence of labelling studies the authors concluded that, after treatment with Tf_2_O, the triflated species **87** is able to interact with LiHMDS in a (formally oxidative) deprotonation event, leading to intermediates **88** or **89**. A second, facile deprotonation by an additional equivalent of LiHMDS then generates the final enamide **86**. This reaction features a broad scope, including straightforward functionalisation of drug molecules.

Slightly prior to this report, Frontier et al. had reported a similar strategy to synthesise spirocyclic isoindolinones **91** from lactams **90** (Scheme 16B).^[36]^ Therein, following triflation of **90**, N‐α‐deprotonation of the generated **92** also results in an oxidative event (sulfinate extrusion), forming **93**. **93**, in turn, tautomerises to cationic enamide **94**, which swiftly undergoes halo‐Nazarov cyclisation to yield the spirocyclic product **91**.

### 2.4 Organocatalytic Electrophilic Amide Activation

**Scheme 17:**
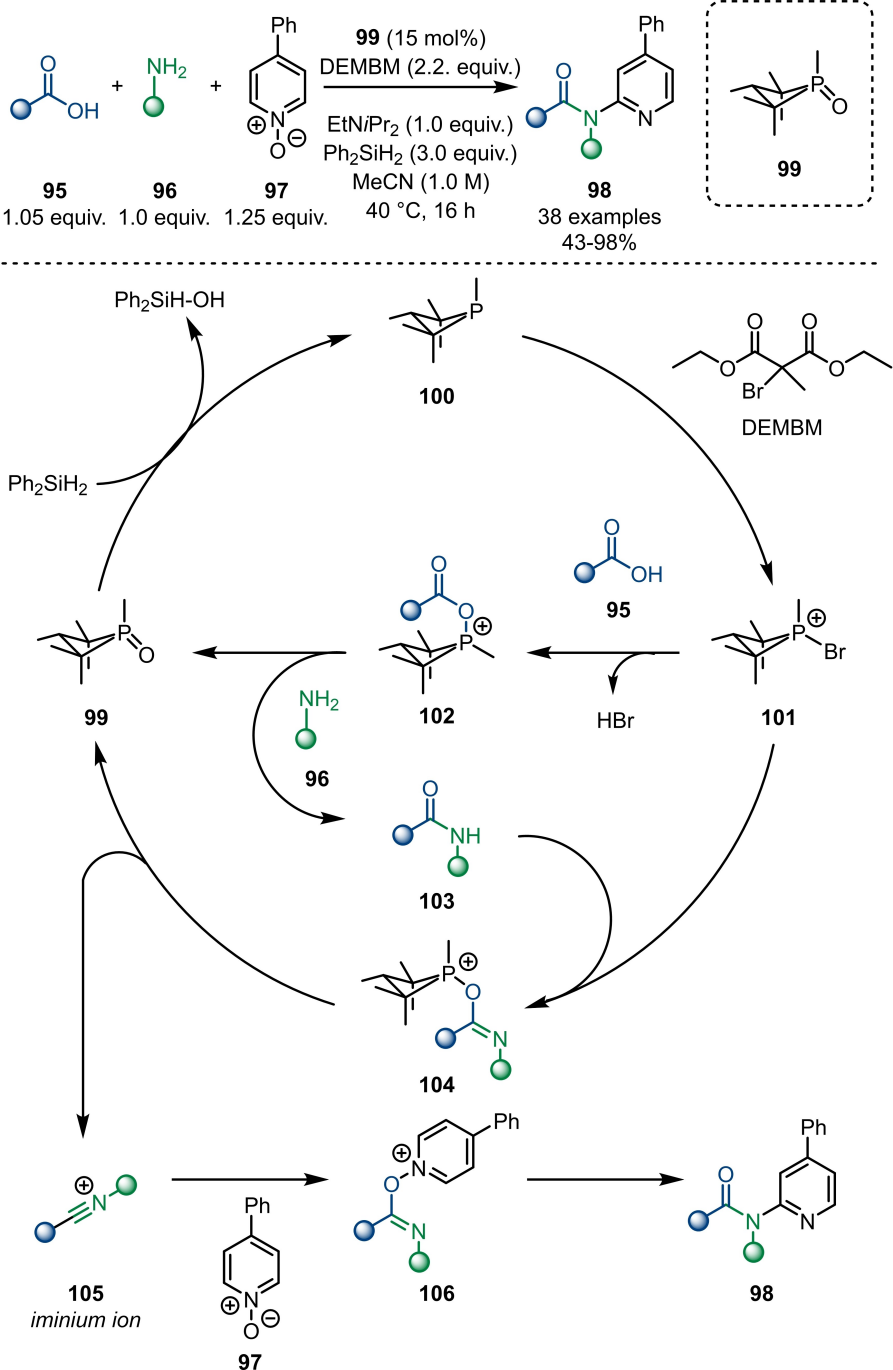
Organophosphorus redox‐catalysed three‐component condensation synthesis of *N*‐pyridyl amides **98**.

Compared to its transition‐metal‐based counterparts, organocatalytic amide activation has been barely explored. Radosevich and Lipshultz recently published an organophosphorus (P^III^/P^V^) redox‐catalysed synthesis of tertiary amides **98** via three‐component condensation of carboxylic acids **95**, amines **96** and pyridine‐*N*‐oxides **97** (Scheme 17).^[37]^ The proposed catalytic cycle is initiated by the reduction of the P^V^‐catalyst **99** with organosilane reagents forming P^III^‐phosphane **100**. Bromination with diethyl (methyl)bromomalonate (DEMBM) forms the reactive intermediate **101**, which acts as an activation reagent for both carboxylic acid **95** and amide **103**. Upon reaction with carboxylic acid **95**, the resulting intermediate **102** is intercepted by amine **96** to furnish amide **103** in situ under regeneration of phosphine oxide **99**. In contrast, the activated amide intermediate **104** fragments to form phosphine oxide **99** and the nitrilium ion **105**, which can be captured by a pyridine‐*N*‐oxide **97** to generate **106**. Further rearrangement and rearomatisation (as already demonstrated in previous work by Abramovitch^[38]^ and Movassaghi^[16]^) leads to tertiary amide **98**. This, to the best of our knowledge, hitherto first organocatalytic strategy might act as the cornerstone for a new generation of electrophilic amide activation approaches.

## 3 Transition‐Metal‐Catalysed Amide Activation

### 3.1 Iridium‐Catalysed Amide Activation

**Scheme 18:**
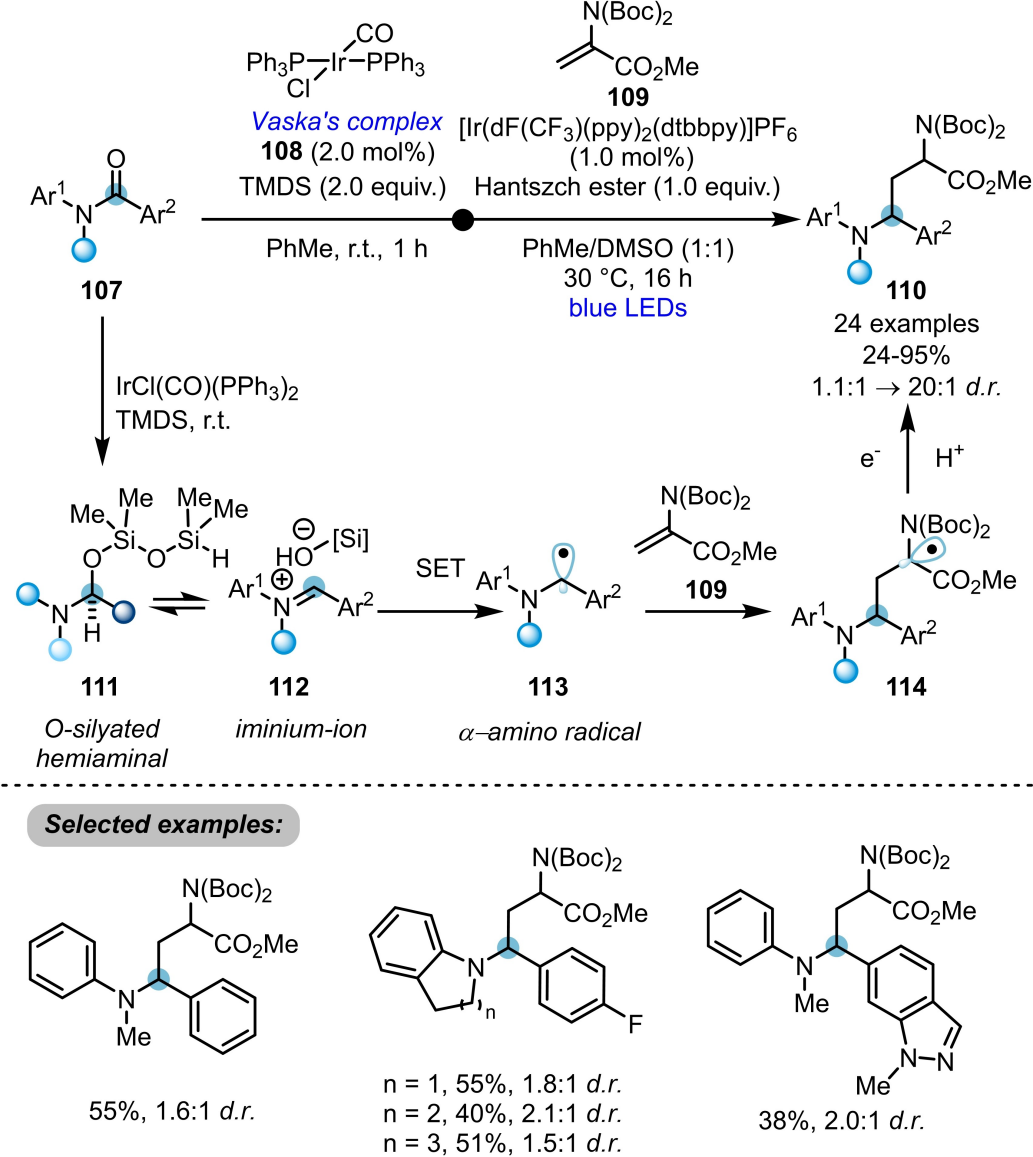
Dual iridium‐catalysed hydrosilylation enabling reductive functionalisation of tertiary amides.

Among the most established strategies for transition‐metal‐catalysed amide activation, IrCl(CO)(PPh_3_)_2_‐(Vaska's complex, **108**)^[39]^‐catalysed hydrosilylation occupies a key place due to its high chemoselectivity for amides in the presence of other functional groups. The Dixon group has been particularly active in exploring the versatility of this catalyst.^[7]^ A prime example of such reactivity can be found in the facile dual iridium‐catalysed reductive functionalisation of tertiary amides **107** via hydrosilylation and single‐electron transfer (Scheme 18).^[40]^ In this transformation, reduction of the amide with Vaska's complex **108** in conjunction with TMDS (1,1,3,3‐tetramethyldisiloxane) smoothly generates iminium ion **112** in equilibrium at room temperature. The concomitant action of photocatalyst and a stoichiometric reductant under visible light irradiation achieves single‐electron reduction to an α‐amino radical intermediate **113**. Following Giese addition of that radical to electron‐deficient alkene **109** and subsequent SET reduction/protonation, the α‐aminoalkylated tertiary amine **110** is formed. A variety of tertiary amine architectures can be prepared in moderate to good yields with typically modest diastereoselectivity.

**Scheme 19:**
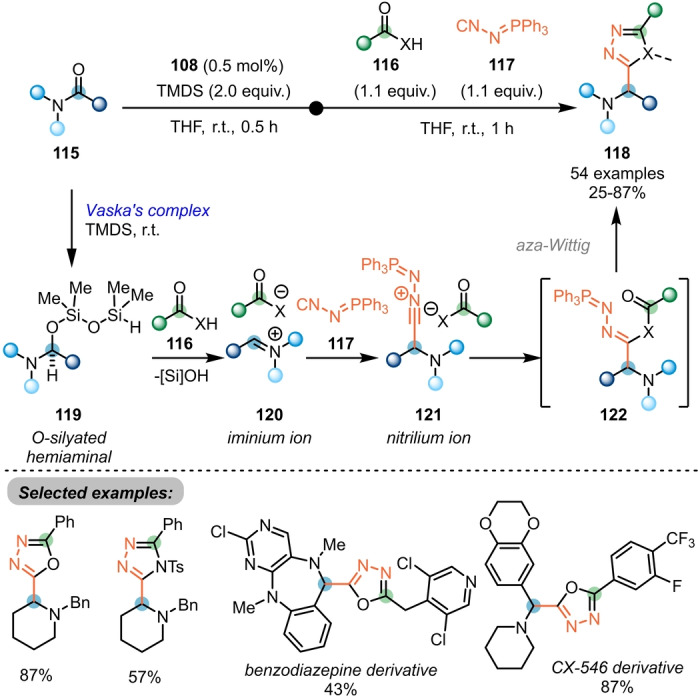
Synthesis of α‐amino 1,3,4‐oxadiazoles and related α‐amino heterodiazole via Ir‐catalysed three‐component coupling of amides with carboxylic acids and NIITP. X=O, S, NTs, NBoc, etc.

Following this report, a three‐component coupling reaction towards the synthesis of α‐amino 1,3,4‐oxadiazoles **118** with tertiary amides **115**, carboxylic acids **116** and (*N*‐isocyanimino) triphenyl‐phosphorane (NIITP, **117**) was reported by the same group (Scheme 19).^[41]^ Initiated by the selective reduction of amides **115** to the iminium ion **120** using Vaska's complex/TMDS, nucleophilic attack of NIITP (**117**) leads to formation of the corresponding nitrilium ion **121**. After conjunction with a carboxylic acid **116** (or an alternative appropriate C‐, S‐, or N‐centred Brønsted acid), direct aza‐Wittig reaction efficiently yields the desired products **118**. This method was successfully applied in the late‐stage modifications of ten drug molecules, thus demonstrating its efficiency. The key intermediate **120** can also be attacked by difluoro‐Reformatsky reagents (BrZnCF_2_R) to afford the medicinally relevant α‐difluoroalkylated tertiary amines, as reported recently by the same group.^[42]^

**Scheme 20:**
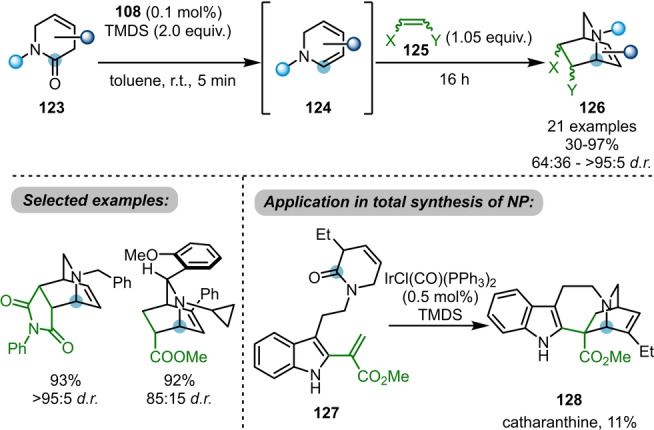
Iridium‐catalysed reductive dienamine generation from lactams to access isoquinuclidines **126** via [4+2]‐cycloaddition. NP=natural product.

The Dixon group further demonstrated that the reduction of β,γ‐unsaturated δ‐lactams **123** with Vaska's complex/TMDS leads to the formation of cyclic dienamines **124** (Scheme 20).^[43]^ These highly reactive species are primed for downstream [4+2]‐cycloaddition reactions with a variety of dienophiles **125** to access bridged bicyclic isoquinuclidines **126** in good yields and diastereoselectivities. This methodology was successfully applied to the total synthesis of catharanthine **128** from the pre‐functionalised indole lactam **127**. Importantly, this approach features high stereocontrol and proceeds with rather low catalyst loading, likely a consequence of the more facile reduction of lactams versus acyclic amides.

**Scheme 21:**
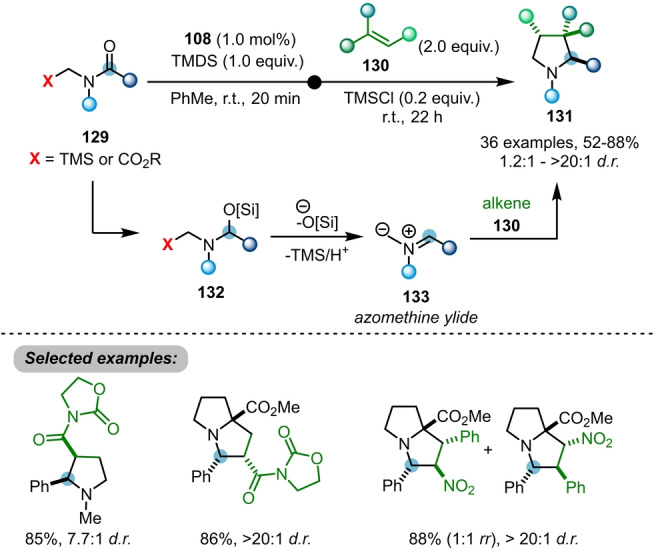
Ir‐catalysed reductive azomethine ylide generation from amides **129**, enabling access to highly functionalised pyrrolidines **131**.

When *N*‐(trimethylsilyl)methyl amides **129** are employed, unstable azomethine ylides **133** are generated in situ via desilylation of **132** (Scheme 21).^[44]^ **133** can be further trapped with dipolarophiles **130** via (3+2)‐cycloaddition to afford highly functionalised pyrrolidines and pyrrolizidines **131**. The reaction proceeds under mild conditions, enabling moderate to high diastereoselectivity and generally good compatibility with a variety of electron‐deficient olefins.

**Scheme 22:**
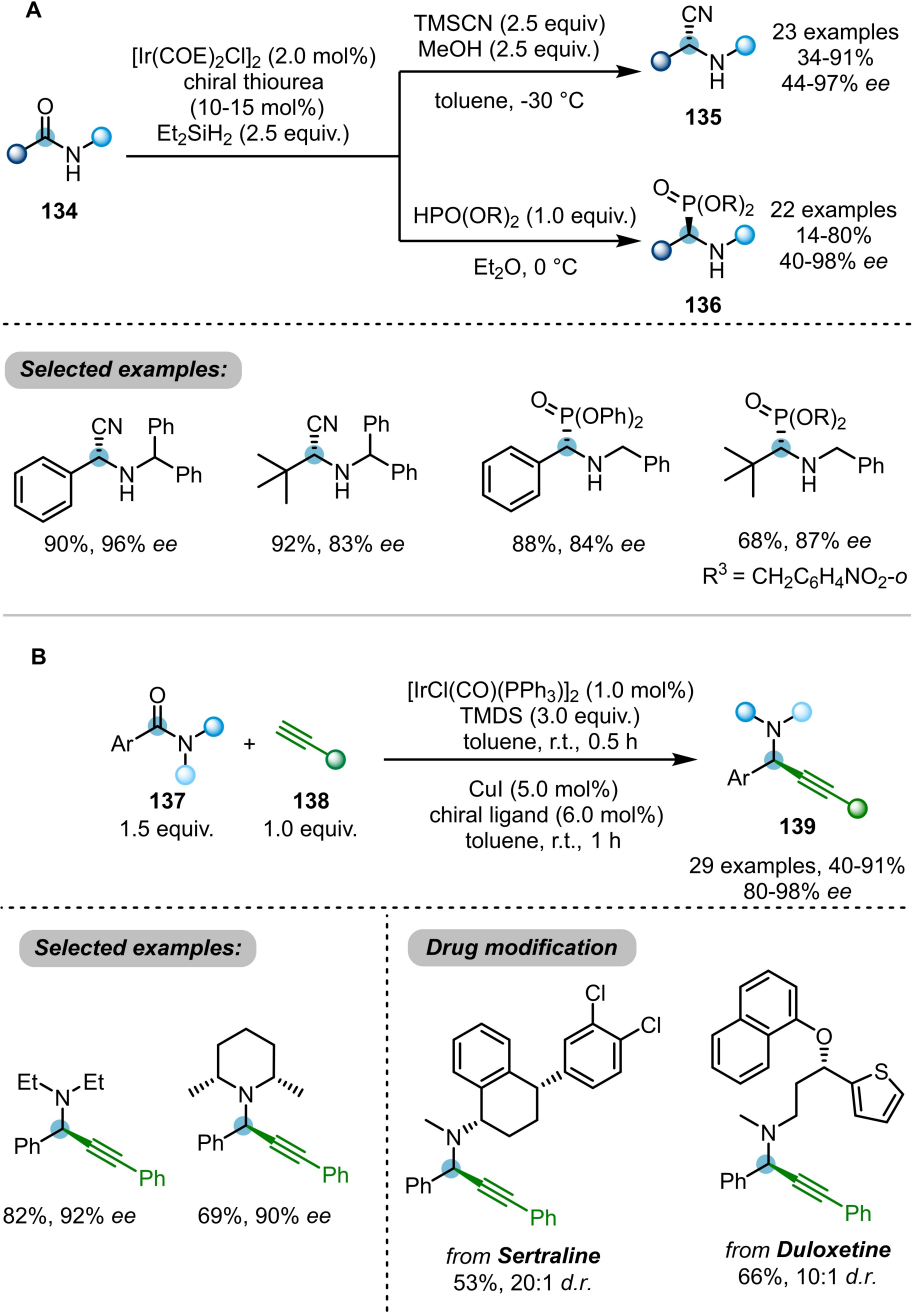
A) Asymmetric reductive cyanation and phosphonylation of amides employing iridium with chiral thiourea. B) Asymmetric reductive alkynylation of amides by iridium/copper relay catalysis.

As shown above, the reduction of amides **115** using Ir^I^‐catalysed hydrosilylation usually affords the electrophilic iminium ion **120** as a key intermediate. By employing different trapping nucleophiles, Huang, Wang et al. were able to capture the iminium ion with different nucleophiles in various asymmetric catalytic systems (Scheme 22A).^[45]^ Enantioselective reductive cyanation and phosphonylation reactions of secondary amides **134** were achieved using trimethylsilyl nitrile (TMSCN) or phosphites as the nucleophiles, in combination with chiral thiourea catalysts. A wide array of enantioenriched chiral α‐aminonitriles **135** and α‐aminophosphonates **136** were prepared using this protocol.

The same groups later reported an asymmetric reductive alkynylation of amides using an elegant Ir/Cu relay catalysis manifold (Scheme 22B).^[46]^ A wide scope of chiral propargylamines **139** were delivered in high yields and *ee* values. Nevertheless, the substrates are limited to the tertiary arylamides.

### 3.2 Nickel‐Catalysed Amide Activation

While, as shown above, iridium can be broadly used to selectively reduce amides to give iminium ions, nickel(0) affords entirely different types of intermediates. The oxidative addition of electron‐rich transition‐metal complexes to destabilised amide C−N bonds leads to metallated intermediates that can be engaged in typical cross‐coupling reactions.[^8^, ^47^] The process of oxidative addition of Ni^0^ to a C−N bond is also involved in a decarbonylation of N‐acylated *N*‐heteroarenes,^[48]^ as well as a cyclisation reaction between aromatic amides and bicyclic alkenes, as recently described by the Chatani group.^[49]^

**Scheme 23:**
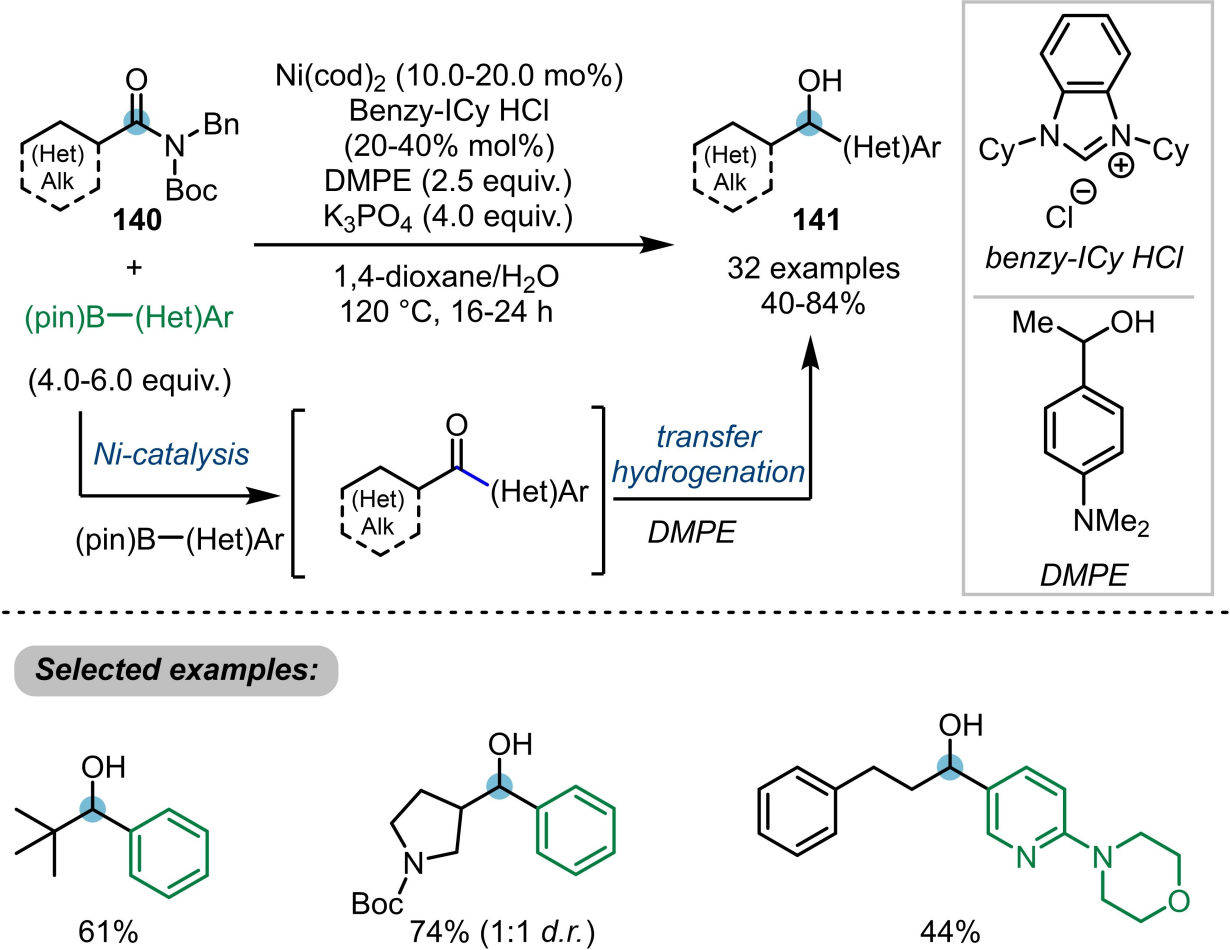
Nickel‐catalysed transformation of twisted amides **140** into alkyl–aryl alcohols **141** via a Suzuki–Miyaura coupling/transfer hydrogenation cascade.

In 2021, Garg et al. developed the first catalytic method for the direct intermolecular addition of two distinct nucleophiles to the carbonyl group of twisted amides **140** (Scheme 23).[^50^, ^51^] Therein, a C−C bond and a C−H bond were formed sequentially, via Suzuki–Miyaura cross coupling followed by transfer hydrogenation, furnishing the secondary alcohols **141** in very good yields.

**Scheme 24:**
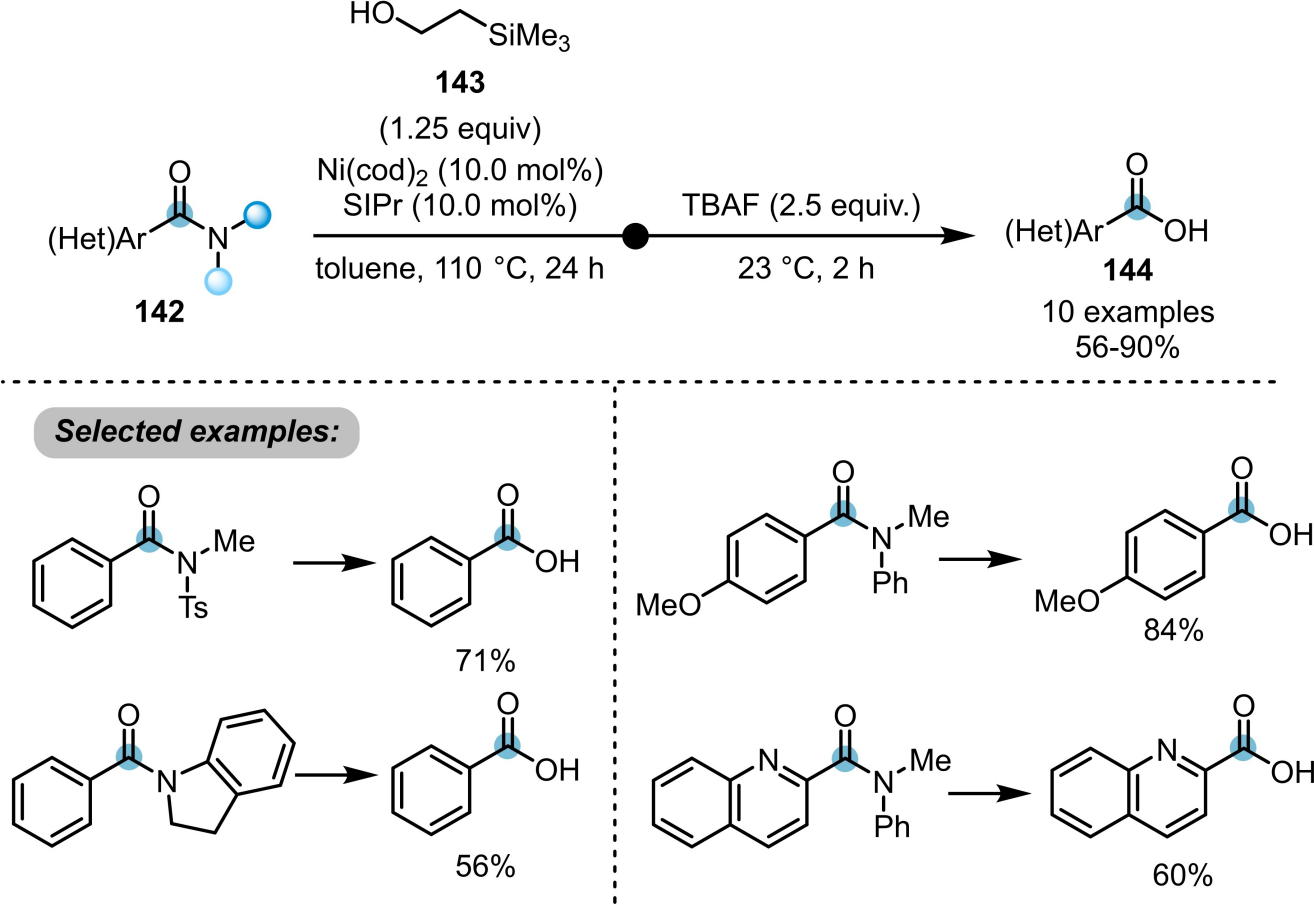
Ni‐catalysed conversion of twisted amides **142** to the corresponding carboxylic acids **144**.

Although a variety of transformations, such as esterification, transamidation and Suzuki–Miyaura cross coupling, was achieved using similar strategies, prior to 2020, no conversion of twisted amides to the corresponding carboxylic acids had been reported.[^8^, ^47^] To remedy this situation, Garg et al. developed an operationally simple procedure, once again employing Ni(cod)_2_ as a pre‐catalyst (Scheme 24).^[52]^ A nickel‐catalysed esterification of benzamides **142** with 2‐(trimethylsilyl)ethanol **143** was first conducted, followed by fluoride‐mediated deprotection in the same pot. Although this method offers a stepwise strategy to convert the tertiary benzamides **142** to the corresponding carboxylic acids **144**, a straightforward transformation of more general amides is still highly required.

### 3.3 Amide Activation Catalysed by Lewis Acids

**Scheme 25:**
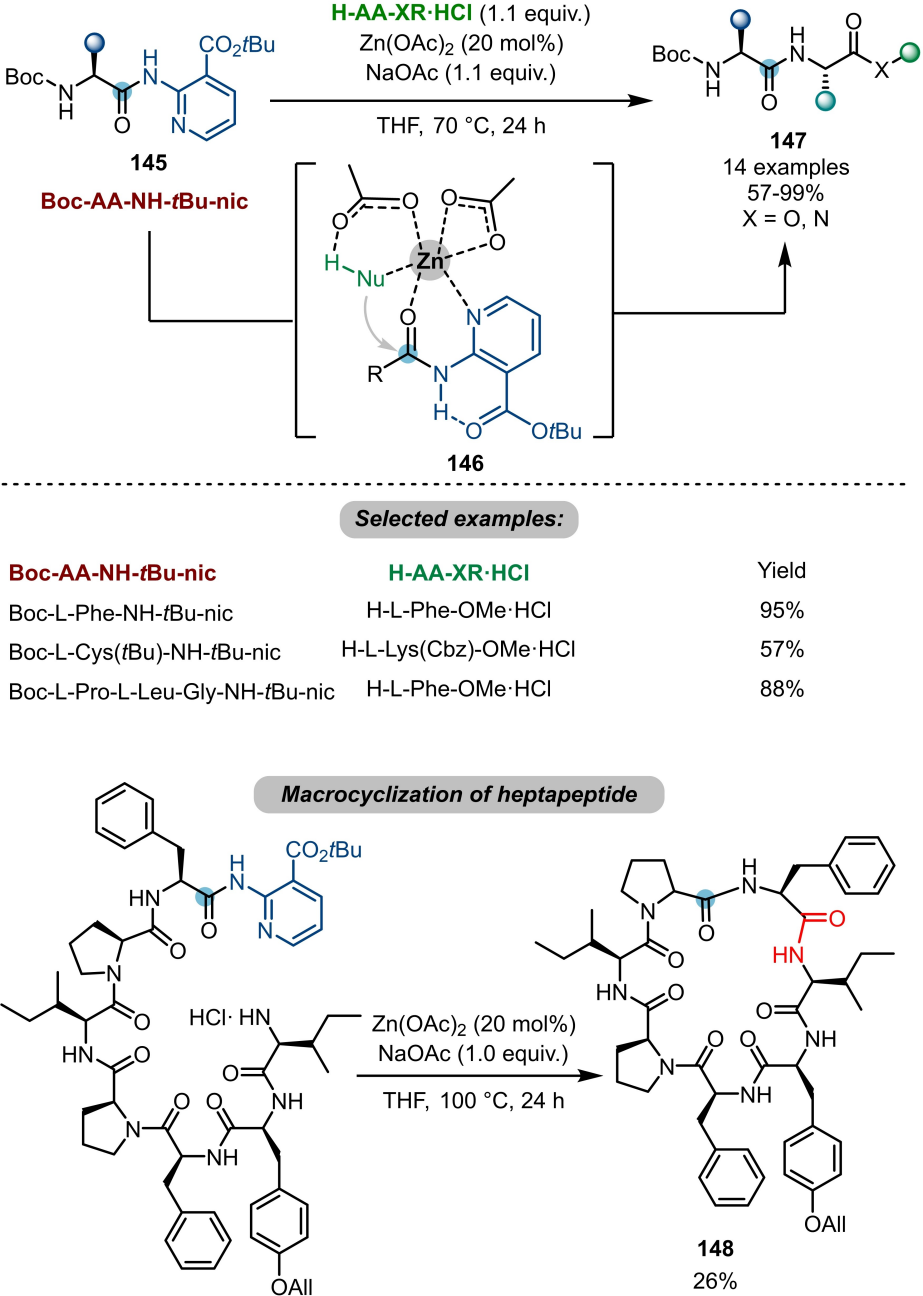
Peptide coupling enabled by zinc‐catalysed transamidations of *t*Bu‐nic‐protected amides **145**.

In a logic similar to that of electrophilic activation, transition‐metal‐based Lewis acids have also been employed to increase the electrophilicity of amides and have allowed subsequent nucleophilic attack. Based on prior work on transesterification,^[53]^ Ballet and Maes et al. achieved a zinc‐catalysed chemoselective transamidation with a *tert*‐butyl nicotinate (*t*Bu‐nic) **145** as a directing group (Scheme 25).^[54]^ Inspired by metallo‐exopeptidases in nature, the authors proposed that the designed directing group (*t*Bu‐nic) might allow bidentate chelation with a transition metal (**146**) and thus assists cleavage of a secondary amide under neutral and mild conditions. Notably, the *t*Bu‐nic‐protected amide **145** is inert under typical peptide coupling/deprotection conditions, which enables its use as a potential building block in peptide synthesis. Furthermore, the authors applied this protocol to a macrocyclisation of a heptapeptide **148**.

**Scheme 26:**
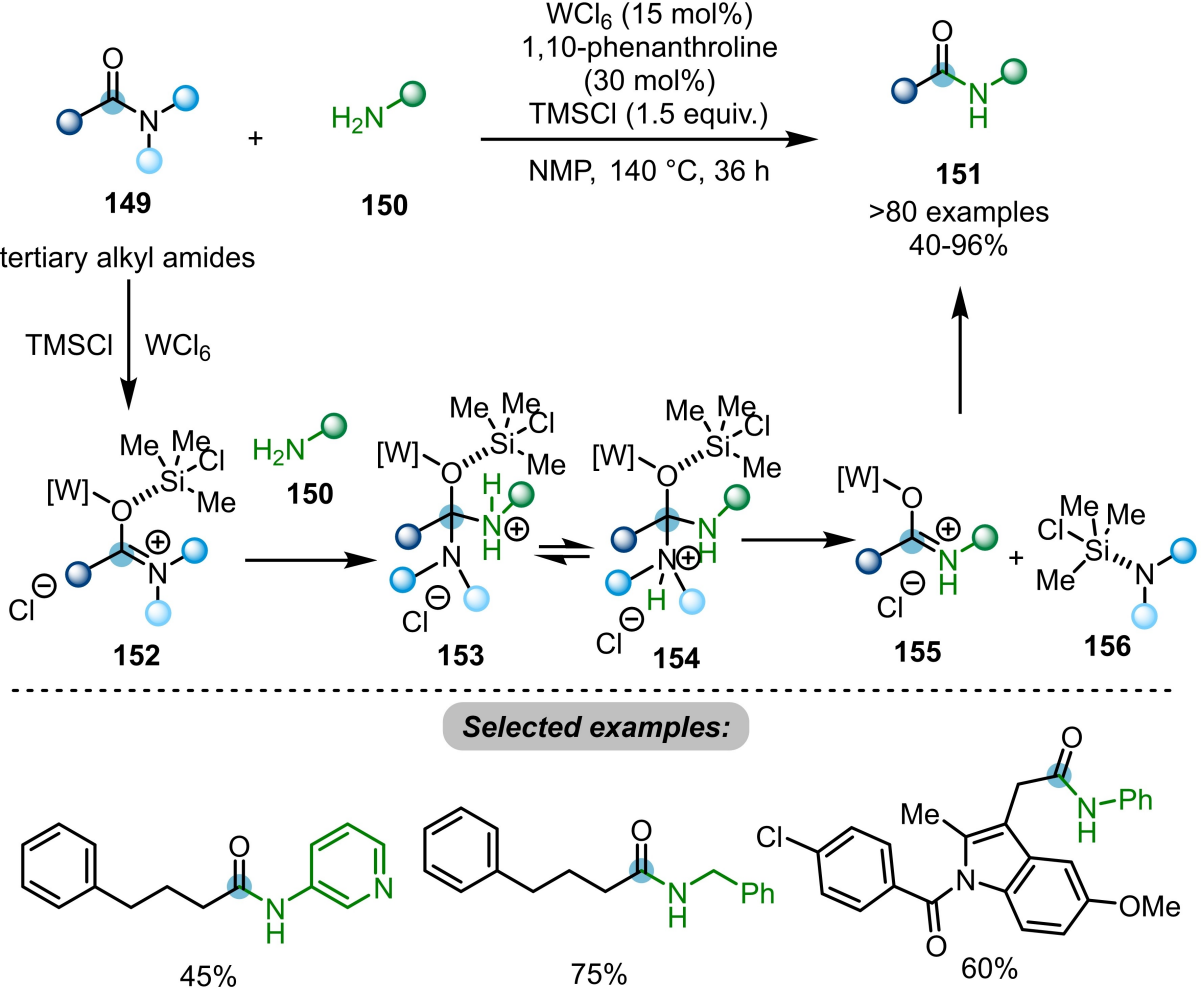
Tungsten‐catalysed transamidation of tertiary alkyl amides **149** to secondary/tertiary amides **151**.

Ma and co‐workers developed a tungsten(VI)‐catalysed transamidation of tertiary alkyl amides **149** to secondary amides **151**, using 1,10‐phenanthroline as ligand with TMSCl as an additive (Scheme 26).^[55]^ In this transformation, the tungsten catalyst activates the amide bond of tertiary alkyl amide **149** to form a cationic O‐bound W–iminium species **152**. Subsequent nucleophilic attack from amine **150** affords the tetrahedral intermediate **153**. Intermediate **154**, which is particularly favourable when a more protic aromatic amine or primary aromatic or alkylamine underwent transamidation, is given via proton transfer. The elimination of more basic dialkyl amine gives **155**, which can further be transformed to the corresponding transamidated product **151**. The authors also showed several limited examples on transamidation of tertiary alkyl amides with secondary amines.

## 4 Other Strategies for Amide Functionalisation

### 4.1 Amide Functionalisations Initiated by Nucleophilic Addition

**Scheme 27:**
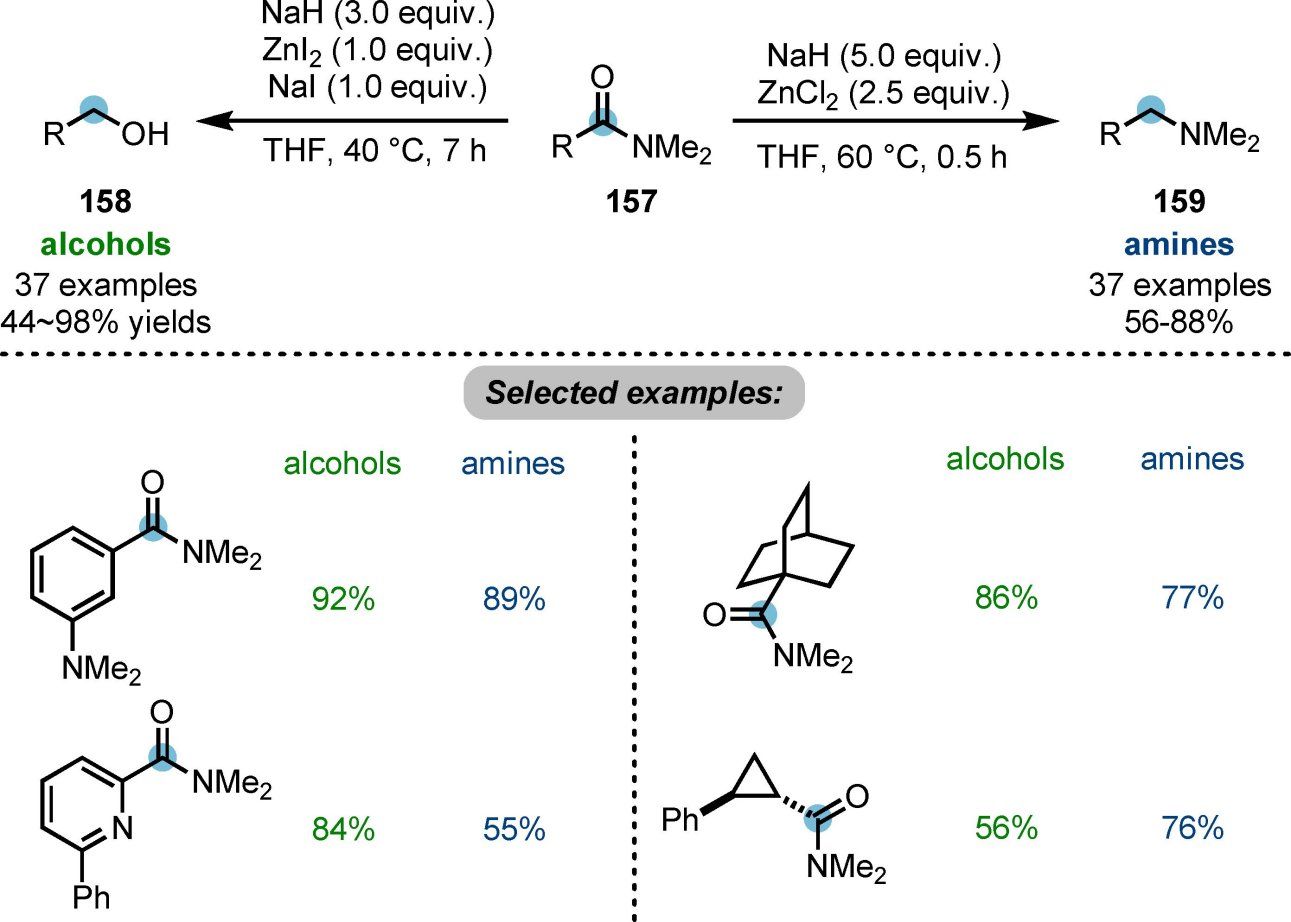
Controlled reduction of carboxamides to alcohols or amines by in situ generated zinc hydrides.

**Scheme 28:**
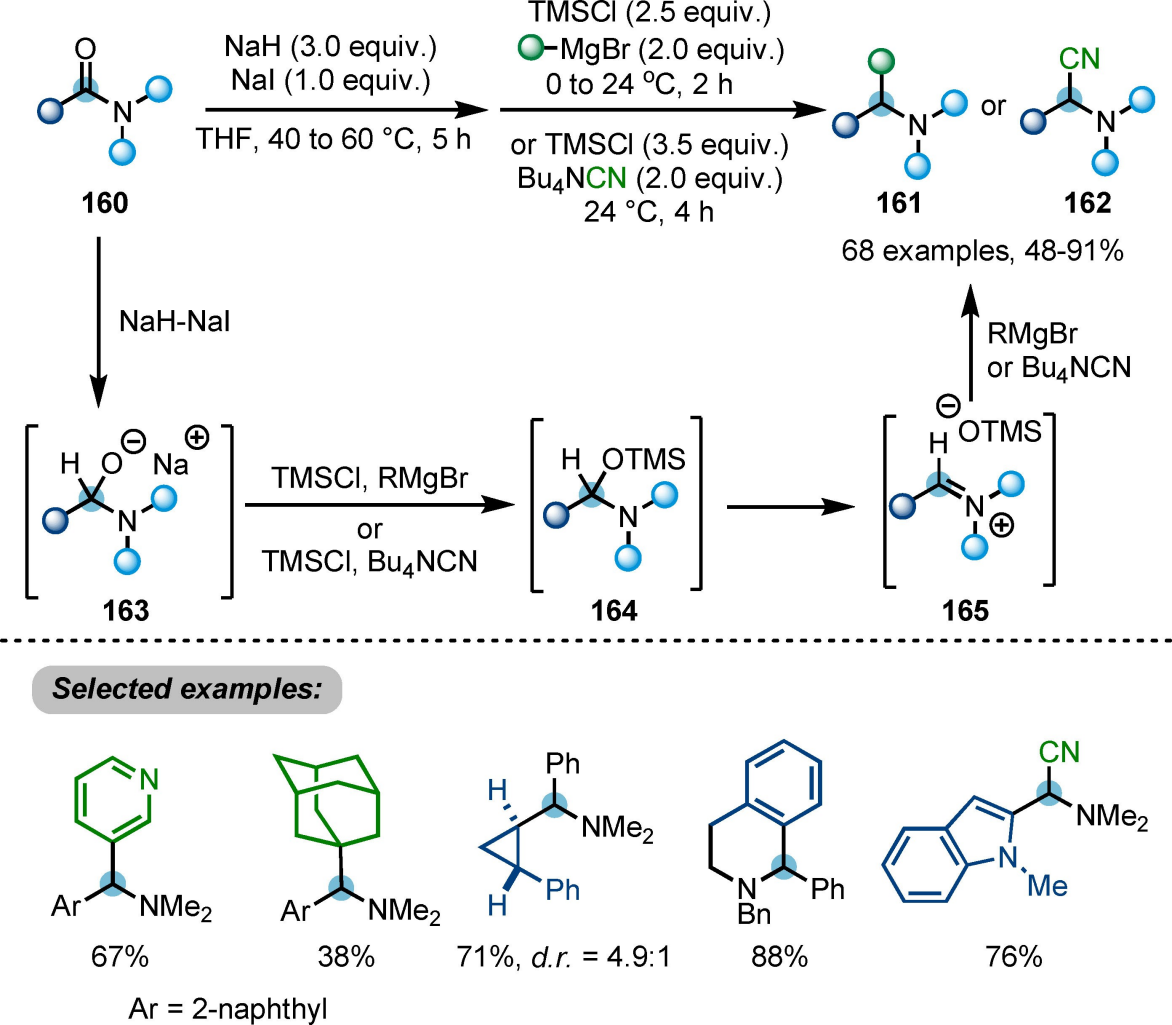
NaH/NaI‐triggered reductive functionalisation of tertiary amides into α‐branched amines **161** and **162**.

In spite of their poorly electrophilic character, amides can still be attacked by strong nucleophiles such as metal hydrides and alkyllithium reagents. The generated tetrahedral intermediate (see **164**, Scheme 28) can be further reduced or functionalised.^[9]^ Chiba et al. developed a strategy for the controlled reduction of carboxamides **157** to alcohols **158** or amines **159** employing zinc hydride, generated in situ by combining sodium hydride (NaH) and zinc halides (ZnX_2_) (Scheme 27).^[56]^ The nature of the halide on ZnX_2_ was shown to afford different zinc hydride species, dictating the selectivity of the reduction. In the NaH−ZnI_2_ system, polymeric zinc hydride (ZnH_2_) is generated, delivering alcohols. On the other hand, the NaH−ZnCl_2_ system forms dimeric zinc chloride hydride (H−Zn−Cl)_2_ and produces amines.

Later, Dixon, Chiba et al. described a reductive functionalisation of carboxamides **160** for the synthesis of α‐branched amines **161** or **162** (Scheme 28).^[57]^ Therein, a stable anionic hemiaminal intermediate **163** is formed through single hydride transfer from the sodium hydride/sodium iodide composite. This species is then captured by TMSCl and the resulting activated hemiaminal **164**. Elimination of trimethylsiloxide generates iminium ion **165**, which can be attacked by other nucleophiles, such as Grignard reagents or cyanide, resulting in deoxygenative installation of a carbon substituent to afford α‐branched amines **161** or **162**. Although a valuable method due to the versatility of the amines that can be synthesised, this transformation is not chemoselective in presence of other carbonyl groups.

**Scheme 29:**
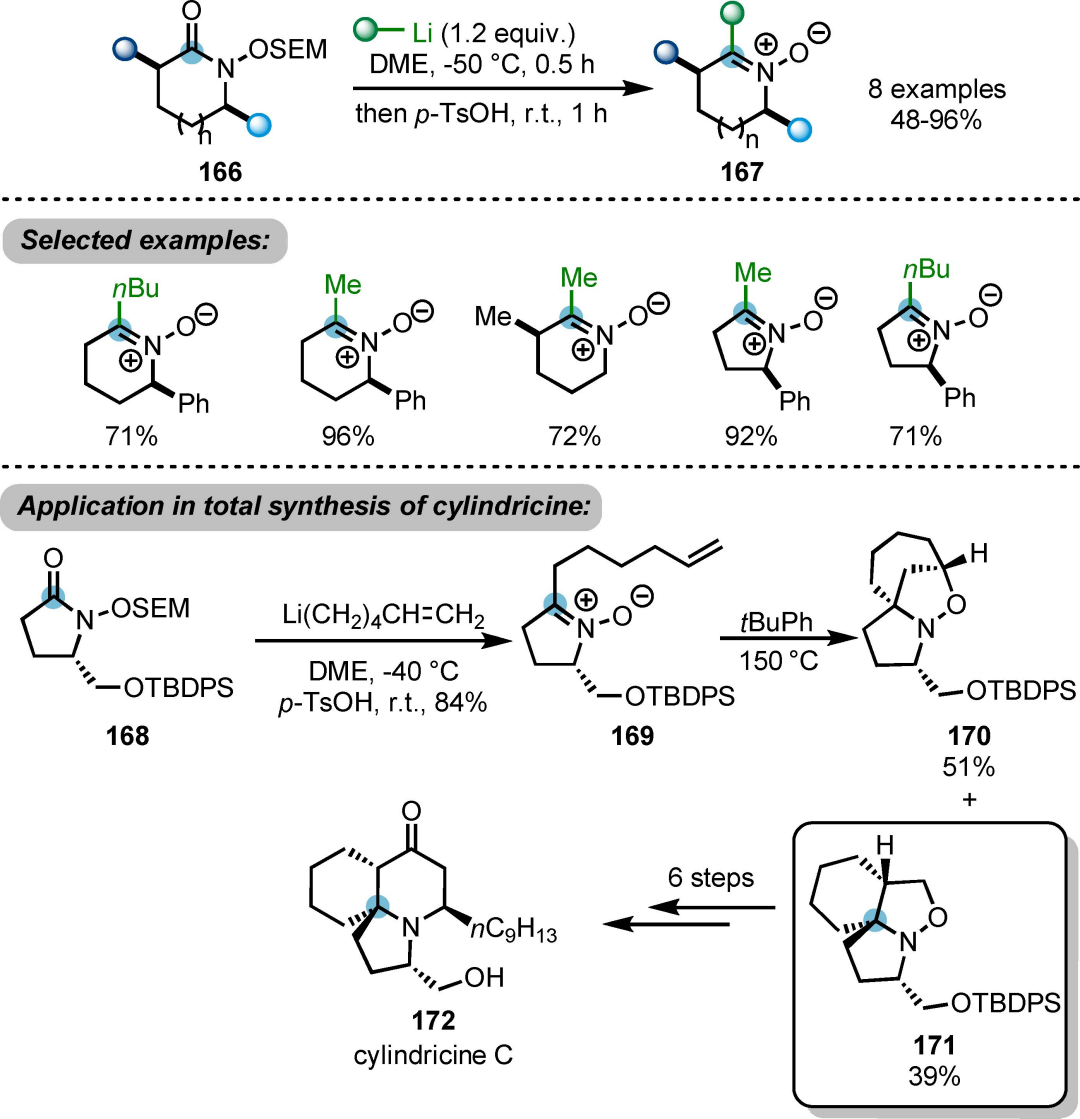
Nucleophilic approach to access highly substituted cyclic nitrones **167** from *N*‐alkoxylactams **166** and its application in the total synthesis of cylindricine C.

Using *N*‐alkoxylactam derivatives **166** as substrates, Sato and Chida et al. developed a useful approach to access highly substituted cyclic nitrones **16**7 (Scheme 29).^[58]^ This process was initiated by the nucleophilic attack of organolithium reagents to **166**, with subsequent removal of the 2‐(trimethylsilyl)ethoxymethyl (SEM) group under acidic conditions, furnishing the desired products **167**. This approach was successfully applied to the total synthesis of cylindricine C **168**. The key chiral nitrone intermediate **169** was afforded with the established method using hexenyl lithium. This intermediate **164** is able to undergo an intramolecular (3+2)‐cycloaddition, giving desired tricyclic product **171** in 39 % yield. The regioselectivity of the cycloaddition step is poor and the major by‐product is the undesired isomer **170**.

**Scheme 30:**
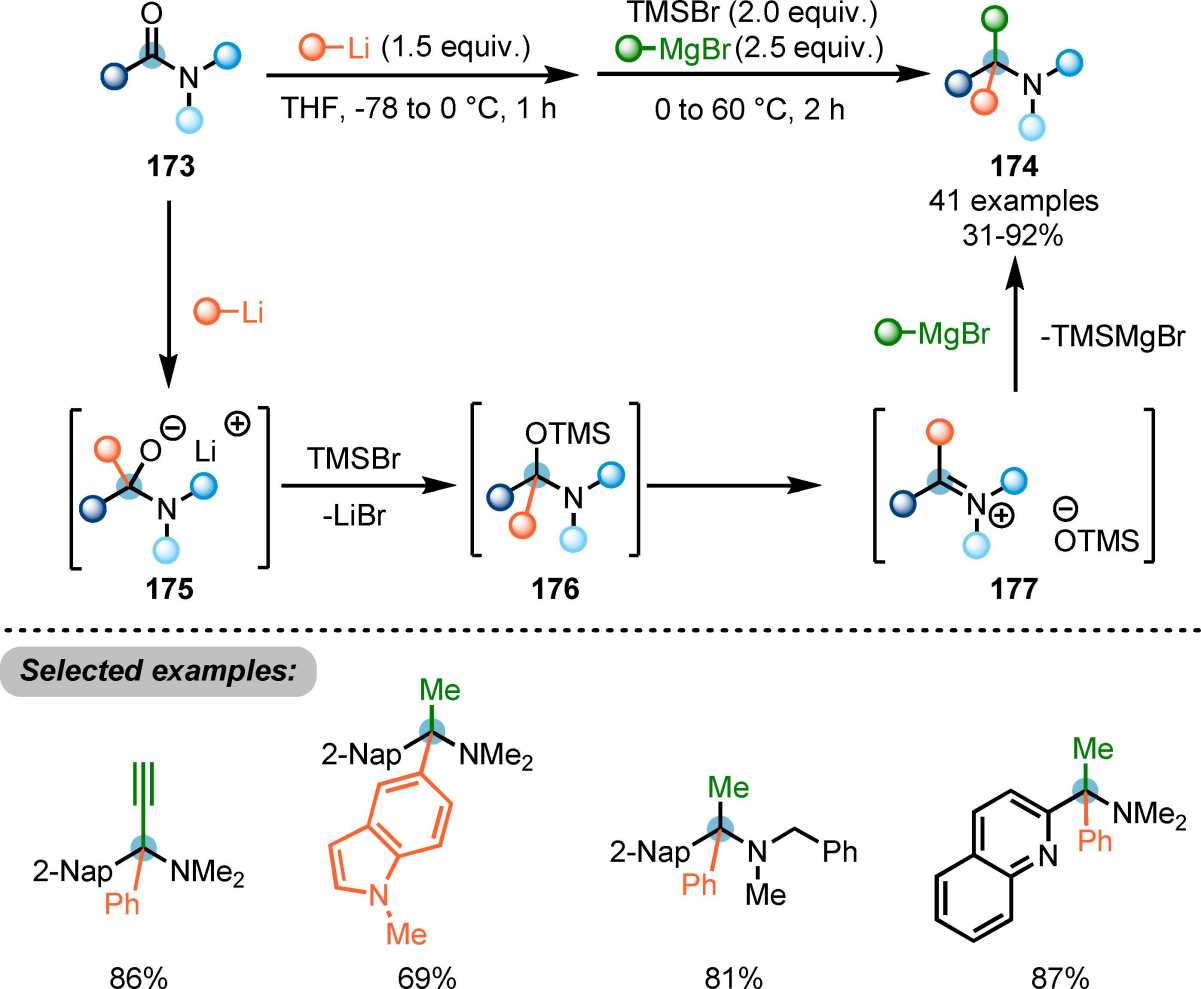
Iterative addition of carbon nucleophiles to *N,N*‐dialkyl carboxamides for the synthesis of α‐tertiary amines **174**.

Recently, Chiba and co‐workers reported a protocol for the synthesis of α‐tertiary amines **174** by iterative additions of carbon nucleophiles to amides (Scheme 30).^[59]^ The proposed mechanism suggested that organolithium addition to the amide carbonyl **173** formed the anionic hemiaminal intermediate **175**, which was detected by NMR analysis. Subsequent in situ O‐silylation gave intermediate **176**. Eventually, the addition of Grignard reagent to the thus formed iminium ion **177** afforded the desired product.

**Scheme 31:**
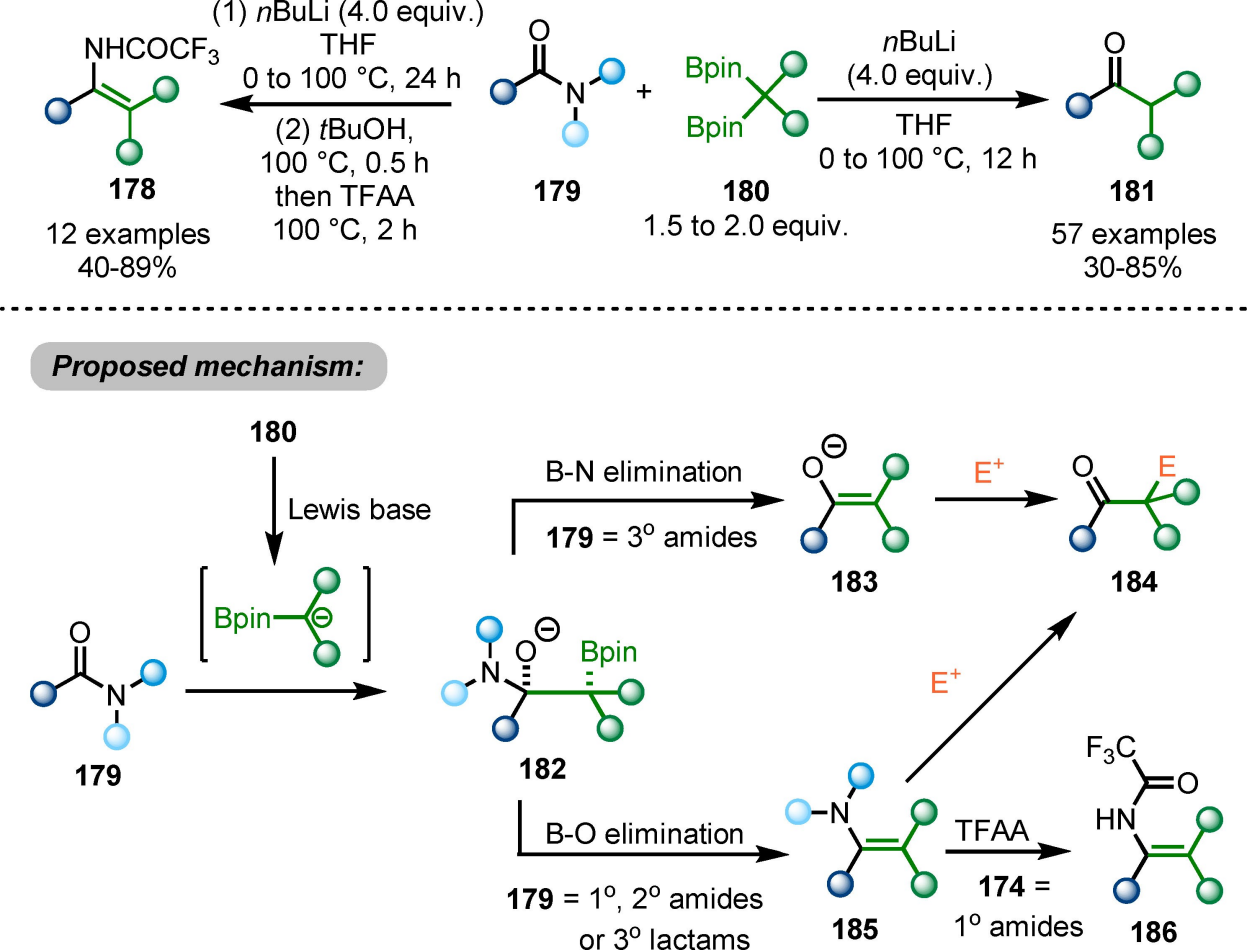
Amide functionalisations by using *gem*‐diborylalkanes as pro‐nucleophiles.

A chemo‐divergent transformation of primary, secondary and tertiary amides, as well as lactams **179**, using 1,1‐diborylalkanes **180** as pro‐nucleophiles was reported by the Liu group in 2020 (Scheme 31).^[60]^ When primary and secondary amides or tertiary lactams are used as the starting materials, B−O elimination of the adduct **182** generates enamine intermediate **185**, while B−N elimination occurs for tertiary amides to generate enolate intermediate **183**. Ketones **184** are afforded via in situ hydrolysis of these intermediates. Trapped by trifluoroacetic anhydride, the enamine intermediates **185** allow the synthesis of enamides **186** from primary amides. This method provides different possibilities of functionalisations of amides. However, the need for an organolithium to activate the 1,1‐diborylalkane pronucleophile might restrict chemoselectivity.

### 4.2 Amide Activation and Functionalisation with SmI_2_/Sm

**Scheme 32:**
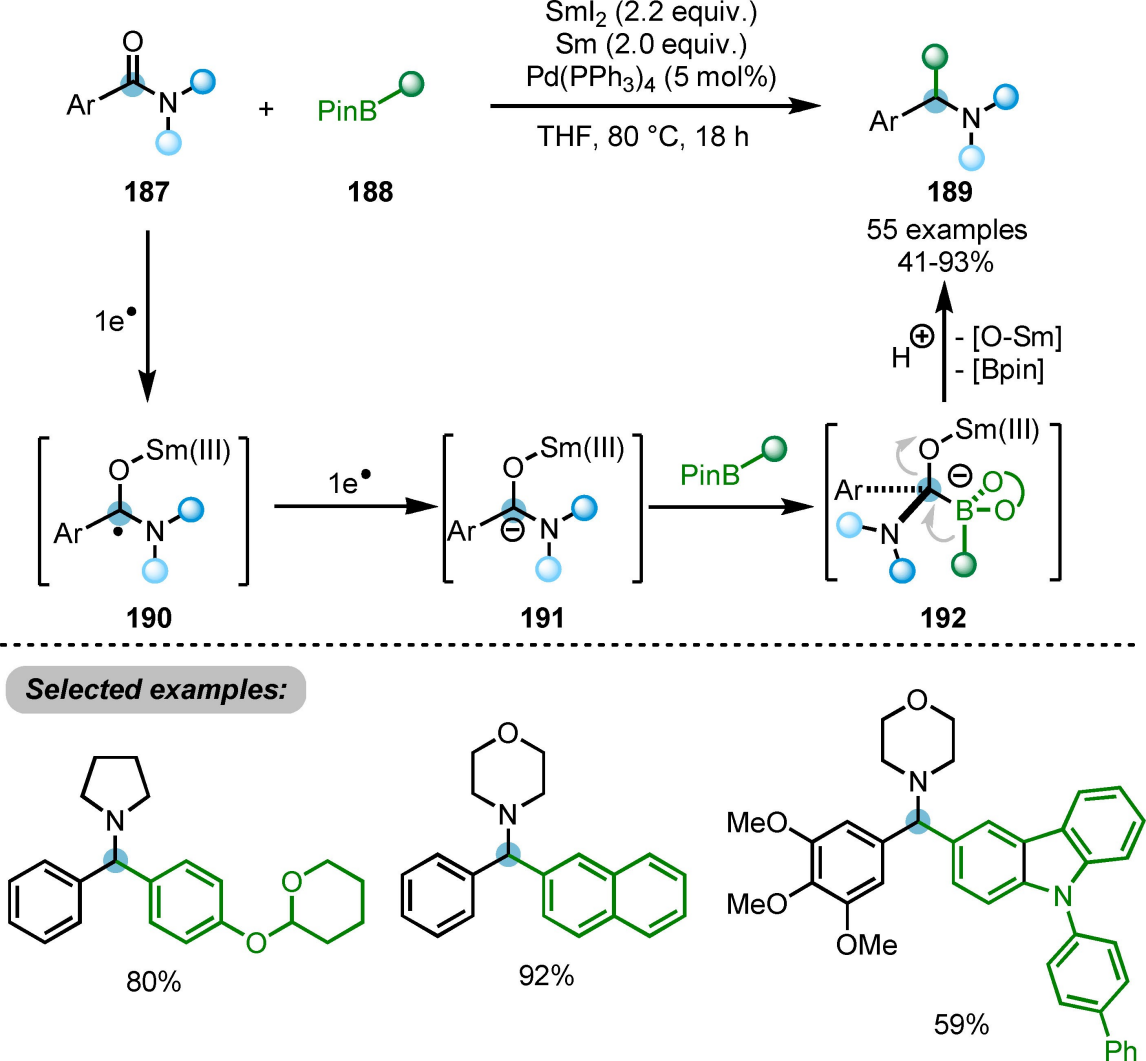
SmI_2_/Sm‐promoted deoxygenative cross‐coupling reaction of amides with arylboronic esters.

**Scheme 33:**
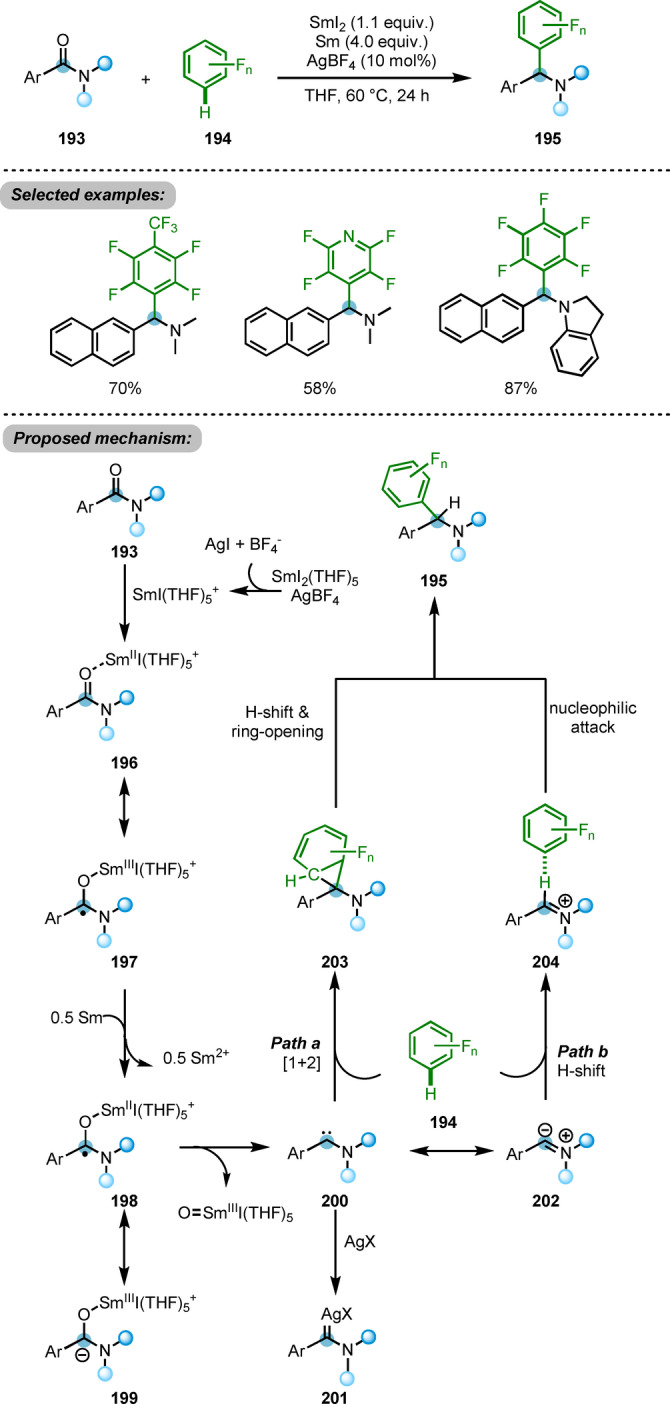
SmI_2_/Sm ‐promoted deoxygenative cross‐coupling reaction of amides with polyfluoroarenes **194**.

Electron transfer to carboxylic acid derivatives mediated by Sm^II^ is a well‐established and powerful strategy to invert the polarity of the carbonyl group, resulting in the formation of a carbon‐centred radical and/or a dianion (Schemes 32 and 33). Wang et al. showed that SmI_2_/Sm‐mediated umpolung of the carbonyl carbon of amide **187** generated an intermediate (**191**) capable of reacting with aryl pinacolboronate ester **188** (Scheme 32).^[61]^ Subsequent boron 1,2‐metalate rearrangement and protodeboronation provided the corresponding arylmethylamines **189** as the products of deoxygenative C−C bond coupling. The authors also showed that the use of catalytic amounts of Pd(PPh_3_)_4_ dramatically increases the yield of the reaction.

A deoxygenative cross‐coupling reaction of amides **193** with polyfluoroarenes **194**, promoted by SmI_2_/Sm, was reported by the same group (Scheme 33).^[62]^ This transformation affords the α‐polyfluoroaryl amines **195** in moderate to good yields through what amounts to a direct C−H functionalisation of polyfluoroarenes **194**. The authors proposed carbon‐centred radical low‐valence Sm^II^ species **198** to be one of the key intermediates of this process. From **198**, abstraction of Sm^III^I‐oxo was proposed to evolve through the cleavage of the C−O bond, forming carbene intermediate **200**, stabilised by the neighbouring nitrogen. **200** could undergo a (1+2)‐cycloaddition with polyfluorobenzene **194** and subsequent 1,2‐hydrogen shift, affording the products **195**. On the other hand, benefiting from the relatively acidic C−H bond of the polyfluoroarene, proton transfer to the iminium intermediate **202** was proposed as an alternative pathway. This cleavage of the C−H bond would form hydrogen bond complex **204**, with subsequent nucleophilic attack of the aryl anion onto iminium providing the desired product **195**. Based on the highly reactive carbene intermediate **200**, other side reactions such as cyclopropanation with alkenes or C−H insertion could be envisioned.

## 5 Conclusion and Outlook

By exploiting the intrinsic electronic and geometric properties of amides, selective amide activation protocols have delivered highly versatile tools to functionalise amides or to convert them into entirely different functional groups. Given the amount of new highly visible publications within the relatively short timeframe covered by this Minireview, it is apparent that amide activation enjoys high interest within the synthetic community. Recent breakthroughs tackled some of the most challenging limitations, such as functionalisation at the “nitrogen part” of amides or the establishment of organocatalytic approaches. Furthermore, new endeavours have been made towards remote functionalisation, stereoselective transformations and overall amide functionalisation strategies. Although important progress has been made, considerable limitations and challenges still exist. First and foremost, there is a clear need to develop even more general approaches. For some of the methods, the reaction scope is limited to specific amides such as tertiary benzamide derivatives. Additionally, while many methods for amide activation are already highly chemoselective, certain transformations still suffer from competition by other carbonyl derivatives or nucleophilic functionality. Notably, the implementation of enantioselective variants remains a highly challenging goal. Considering the recent breakthroughs and the current limitations, amide activation offers a vast field for additional explorations further challenging the conventional, by now perhaps outdated, view of this functional group.

## Conflict of interest

The authors declare no conflict of interest.

## Biographical Information

*Minghao Feng received his Ph.D. in 2017 from the East China Normal University, working on the methodology‐oriented total synthesis of alkaloids, supervised by Prof. Xuefeng Jiang. Funded by a Marie‐Curie Eurotalent Fellowship, he worked on click chemistry and drug isotope labelling with Dr. Frédéric Taran and Dr. Davide Audisio as a postdoctoral researcher at CEA‐Saclay from 2018 to 2020. He is now a Lise‐Meitner senior postdoctoral fellow in the group of Prof. Nuno Maulide, pursuing the development of asymmetric methodologies based on amide activation*.

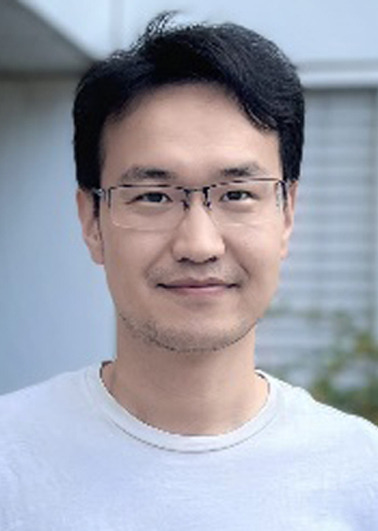

## Biographical Information

*Haoqi Zhang is a Ph.D. student working in the group of Prof. Nuno Maulide at the University of Vienna. He received his MSc degree in the same group in 2020, having worked on electrophilic umpolung of amides to construct novel heterocycles. For his Ph.D. thesis, he primarily dedicates his work to the synthesis of drug candidates and natural product‐like structures, using approaches focusing on challenging cyclisations*.

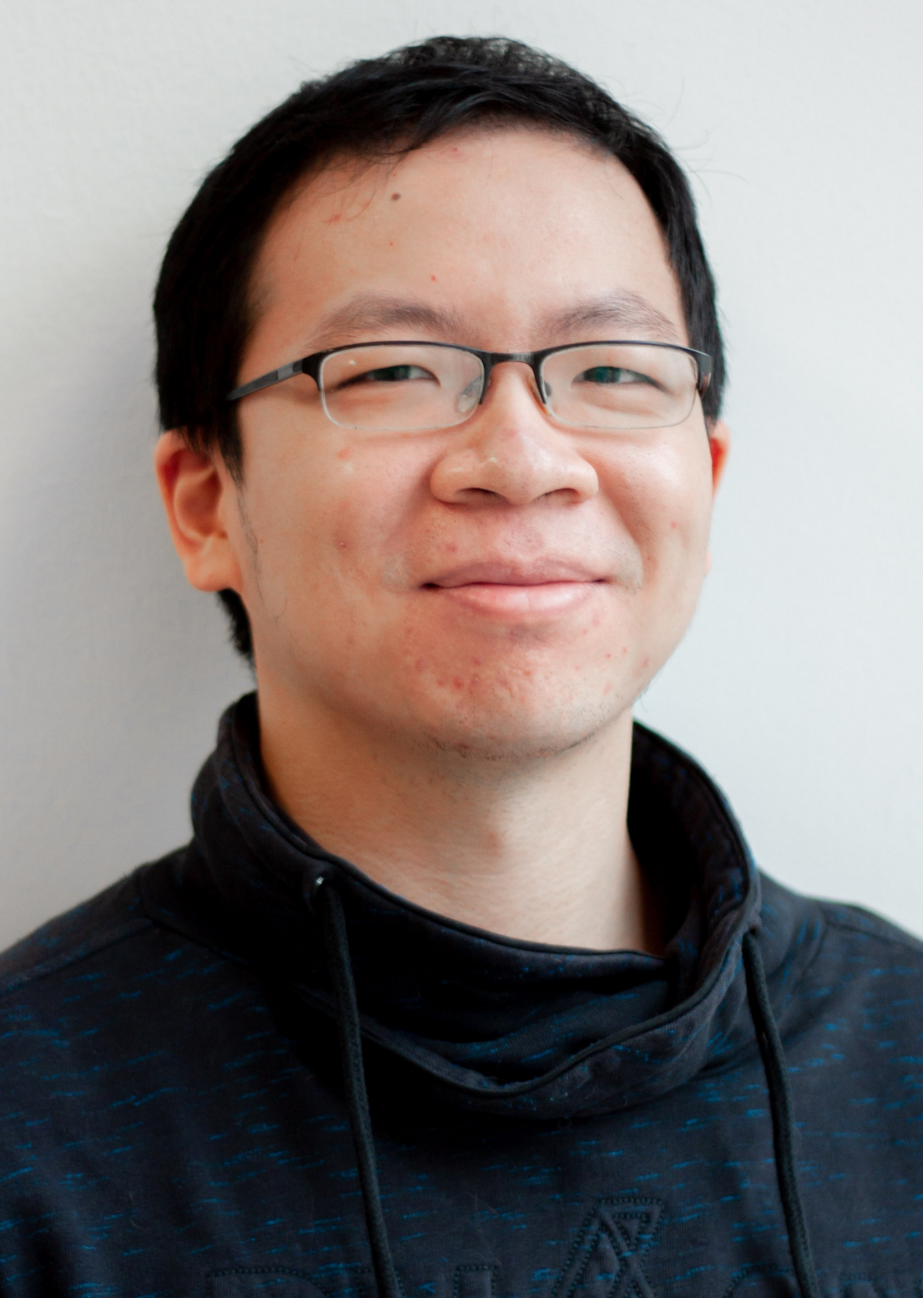

## Biographical Information

*Nuno Maulide is Full Professor of Organic Synthesis at the University of Vienna (Austria) since 2013. His research focuses on the exploration of unconventional reactivity profiles in organic chemistry and has been acknowledged by several awards (including Austria's Scientist of the Year 2019, Tetrahedron Young Investigator Award 2020 and election as Full Member of the Austrian Academy of Sciences in 2021)*.